# UAV Multisensory Data Fusion and Multi-Task Deep Learning for High-Throughput Maize Phenotyping

**DOI:** 10.3390/s23041827

**Published:** 2023-02-06

**Authors:** Canh Nguyen, Vasit Sagan, Sourav Bhadra, Stephen Moose

**Affiliations:** 1 Taylor Geospatial Institute, St. Louis, MO 63108, USA; 2 Department of Earth and Atmospheric Sciences, Saint Louis University, St. Louis, MO 63108, USA; 3 Department of Aviation, University of Central Missouri, Warrensburg, MO 64093, USA; 4 Department of Crop Science and Technology, University of Illinois, Urbana, IL 61801, USA

**Keywords:** UAV, data fusion, multi-task deep learning, high-throughput phenotyping, hyperspectral, LiDAR, GeoAI

## Abstract

Recent advances in unmanned aerial vehicles (UAV), mini and mobile sensors, and GeoAI (a blend of geospatial and artificial intelligence (AI) research) are the main highlights among agricultural innovations to improve crop productivity and thus secure vulnerable food systems. This study investigated the versatility of UAV-borne multisensory data fusion within a framework of multi-task deep learning for high-throughput phenotyping in maize. UAVs equipped with a set of miniaturized sensors including hyperspectral, thermal, and LiDAR were collected in an experimental corn field in Urbana, IL, USA during the growing season. A full suite of eight phenotypes was in situ measured at the end of the season for ground truth data, specifically, dry stalk biomass, cob biomass, dry grain yield, harvest index, grain nitrogen utilization efficiency (Grain NutE), grain nitrogen content, total plant nitrogen content, and grain density. After being funneled through a series of radiometric calibrations and geo-corrections, the aerial data were analytically processed in three primary approaches. First, an extended version normalized difference spectral index (*NDSI*) served as a simple arithmetic combination of different data modalities to explore the correlation degree with maize phenotypes. The extended *NDSI* analysis revealed the NIR spectra (750–1000 nm) alone in a strong relation with all of eight maize traits. Second, a fusion of vegetation indices, structural indices, and thermal index selectively handcrafted from each data modality was fed to classical machine learning regressors, Support Vector Machine (SVM) and Random Forest (RF). The prediction performance varied from phenotype to phenotype, ranging from *R*^2^ = 0.34 for grain density up to *R*^2^ = 0.85 for both grain nitrogen content and total plant nitrogen content. Further, a fusion of hyperspectral and LiDAR data completely exceeded limitations of single data modality, especially addressing the vegetation saturation effect occurring in optical remote sensing. Third, a multi-task deep convolutional neural network (CNN) was customized to take a raw imagery data fusion of hyperspectral, thermal, and LiDAR for multi-predictions of maize traits at a time. The multi-task deep learning performed predictions comparably, if not better in some traits, with the mono-task deep learning and machine learning regressors. Data augmentation used for the deep learning models boosted the prediction accuracy, which helps to alleviate the intrinsic limitation of a small sample size and unbalanced sample classes in remote sensing research. Theoretical and practical implications to plant breeders and crop growers were also made explicit during discussions in the studies.

## 1. Introduction

Timely and accurate crop estimates prior to harvest have a great impact on national food policy [1], food security, and personal living standards [2]. The conventional estimation, however, has heavily relied on ground-based field surveys, which are labor-costly and prone to poor crop assessment [3]. Therefore, developing a low-cost, rapid, and accurate high-throughput method for phenotyping at a field scale is acutely desired for crop production. Recent technological advancements in unmanned aerial vehicles (UAV) and sensor miniaturization have filled the current explosive demand for precision agriculture in general and for high-throughput plant phenotyping in particular. With a UAV system, aerial data at very fine and high spectral, spatial, and temporal resolutions can be remotely acquired over small to medium fields for crop monitoring in cost-efficient and rapid flight missions [4,5]. The choice of UAV is generally not a matter when both fixed- and rotary-wing can carry automated phenotyping tasks; the matter rests in the payload and mounted sensors that would dictate the purpose of the study.

Countless previous studies conducted unmanned aerial missions to scout various crops: soybean [5], corn [6], sunflower [7], rice [8], maize [9], cotton [10], but most of them exploited crop properties from passive remote sensing data recorded on a few to several spectral wavelengths such as red–green–blue (RGB) and multispectral sensors mounted on UAV platforms. Fewer studies used UAV-based hyperspectral imaging (HSI) in plant phenomics including biochemical traits: chlorophyll [11,12], nitrogen [13], biophysical traits: biomass [12,14], height and leaf area index (LAI) [12]), physiological traits (water status [15], stomatal conductance and fluorescence [16]), biotic stress (i.e., disease) [17,18], and grain yield [19,20,21,22]. Its broad applicability is perhaps because that hyperspectral imaging increases the wavebands to hundreds and even thousands of contiguous spectra in both visible (VIS) and near-infrared (NIR) regions, which provides enriched pertinent spectral information of objects. As an imagery cube, it concurrently offers spatial information along the image height and width, as well as continuous spectral information along the image depth.

To a certain extent, the great abundance of information of hyperspectral cubes poses a variety of challenges in processing and interpreting the data. The imbalance between the high dimensionality of the imagery data and the limited availability of training samples often occurs in remote sensing datasets, which is also known as the Hughes phenomenon [23]. An adoption of dimensionality reduction strategies is necessary to alleviate the issue, including but not limited to selecting a set of wavelengths [24,25,26], handcrafting representative features such as vegetation indices [16,18], orthogonal transformation (e.g., principal component analysis) [27], derivative analysis [24], and wavelets and correlation plots [28]. Preserving the great richness of hyperspectral images is a strenuous task if one approaches the process on an image-wise basis, as the nature of spatial–pectral information varies among inter- and intra-objects in a scene of view. Machine vision with widely known techniques, convolutional neural networks (CNNs) and its variants in 1D-CNNs, 2D-CNNs, 3D-CNNs, or hybrid, could automate the task by sliding kernel patches to obtain both spatial and spectral representations for regression or classification prediction. The extraction of interrelated spatial–spectral features can be done by two common methods. It can process the spatial features separately by 1D-CNNs or 2D-CNN [29,30] and then incorporate the resulting spatial features with the spectral features extracted from the Recurrent Neural Network (RNN), Long Short-Term Memory (LSTM) [30,31] to have a complete fusion. It can be alternatively done by leveraging 3D-CNNs [18] with 3-dimensional patches (*p* × *p* × *b*) associated with *p* × *p* spatial neighborhood pixels and b spectral bands to extract spatial in tandem with spectral abstracts, which fully exploits important discriminative patterns in the hyperspectral data cubes. This is not to mention that the challenges are exponentially amplified by the working complication of the UAV hyperspectral system when a moving UAV platform and the maneuvering offsets must be taken during the imagery calibration process [32]. The following sections in this study will address these challenges in detail from various angles.

UAV thermal (TIR) are other passive optical remote sensing data, ranging at 3–14 µm in the electromagnetic spectrum. The aerial thermal platform is simply and cost-effectively operational and thus, has been widely used in monitoring terrestrial vegetation via measures of canopy temperature and spectral emissivity [33]. The aerial thermal imaging has been introduced as a very versatile tool for various applications: for instance, discerning crop water stress status [34,35,36], irrigation scheduling [37]. In regard to plant phenotyping, thermal imaging remained underexploited [38,39] in spite of its potentials. Spectral attributes from visual (VIS) and near infrared (NIR), or even short-wave infrared (SWIR) regions are inadequate for capturing polysaccharides components such as cellulose and leaf surface properties including waxes and hairs, which are mainly reflected on the TIR domain [40]. This fact suggests that the UAV thermal could be a complement to spectral sensing and thus deliver more accurate phenotype estimations. Only [41] showed the effectiveness of a combination between thermal and multispectral features in predicting nitrogen concentration and chlorophyll *a* (Chl a) content. In our study, the singularity of thermal imaging and the fusion with spectral imaging will be processed both feature-wise and image-wise under the framework of CNNs.

Light detection and ranging (LiDAR) is an active remote sensor that can rapidly and precisely record 3D structural characteristics of terrestrial vegetation in a formation of backscattering points (a.k.a. point clouds). Unlike optical remote sensing, airborne LiDAR sensed information has less relation to photosynthetic scheme of crops, but is able to detail canopy closure patterns, canopy height, and leaf angle distribution that affect the forming of crop traits. The low-altitude airborne sensor has been successfully used in many agricultural applications, such as canopy height [42], tree species classification [43,44], land use land cover [45], and crop biomass-related traits such as above ground biomass [46,47]. It should be noted that in addition to height-associated factors, LiDAR also offers point intensity, which is a measure, collected for every point, of the return strength of the laser pulse that generated the point. It is based, in part, on the reflectivity of the object struck by the laser pulse. Other descriptions for intensity include return pulse amplitude and backscattered intensity of reflection that is a function of the near-infrared wavelength used in the LiDAR. Intensity is used as an aid in feature detection and extraction, in lidar point classification, and as a substitute for aerial imagery when none is available. The contribution of UAV LiDAR intensity to high-throughput plant breeding is unknown. This study was conducted to provide insights about the potential of airborne LiDAR sensing towards crop monitoring.

It is ideal if those above-discussed data sources become intermingled by some means of data fusion that then benefit crop estimations at a higher accuracy. Several recent studies proved this pathway at a certain confidence level in classifying forest tree species [48,49], detecting pine wilt disease [50], and estimating crops’ traits such as grain yield [5] and seed compositions [51]. Among these works, very few exploited the full potential of deep learning and convolutional neural networks, in particular, for aerial multisensory data fusion. Adding to the further side of a more accurate multimodal fusion model, a multi-task deep learning model consuming multiple data modalities to predict multiple crop phenotypes simultaneously is strongly desired to surpass and has not even existed in the literature.

To fill the research gap presented above, the overarching objective of this research was to explore the possibility of UAV remote sensing being instrumental for high-throughput phenotyping in maize by deploying airborne multisensory data fusion with a single multi-task deep learning model. To address it, we aimed to achieve the following sub-objectives: (1) developing a machine learning model for multisensory data fusion of very high-resolution UAV borne hyperspectral images, thermal images, and LiDAR point clouds to estimate a full suite of maize phenotypic traits, (2) assembling an end-to-end multimodal fusion multi-task deep convolutional neural network in a phenotyping regression context, (3) examining the individual and fused contributions of each data multimodality to a range of maize trait predictions, and (4) evaluating the impact of data augmentation on the multimodal fusion multi-task deep learning regression to address a limited sample size in remote sensing research.

## 2. Materials and Preprocessing

### 2.1. Test Site and UAV Data Acquisition

**Figure 1:**
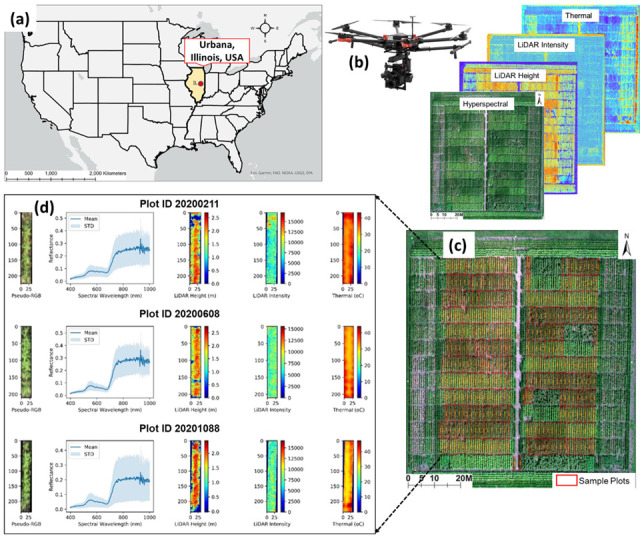
Experiment site location (red dot) in Urbana, Illinois, USA (**a**), UAV aerial hyperspectral, LiDAR, and thermal data collected from the field (**b**), the ortho-mosaic of maize field and samples plots (red polygons) (**c**), UAV hyperspectral, LiDAR height, LiDAR intensity, and thermal data of random plots enlarged and visually projected (**d**).

An experimental corn field was set up between early May and late September in 2020 at the Crop Sciences Research and Education Center located near the University of Illinois campus in Urbana, IL, USA (40.08 N, 88.22 W) (Figure 1a). The corn field has a north–south dimension of 93 m and 32.6 m in the east–west dimension. The experiment was organized in three areas: north–south edges, east–west edges, and the center field. On the north and south edges, a block of 8 rows with 4 inside rows of genotype ILO3 × ILP1 and 4 outside rows of commercial hybrids were grown as a cross border. The east and west edges were grown with 29 corn inbred genotypes in single row plots. The main center field, which was a focal interest of this study, was an experiment of a collection of 66 corn hybrid genotypes representing two populations, diversity and high-nitrogen response. The experimented soil type was a Drummer silty clay loam with 6.5 pH that was equivalent to a source of 60 kg nitrogen per hectare estimated by subsequent soil sampling and measures of plant nitrogen recovery. A primary treatment exposed maize blocks with either no supplemental nitrogen (low N) or nitrogen fertilizer (high N) at a rate of 225 kg/ha as granular ammonium sulfate (AMS) at the soil surface. The nitrogen fertilization was randomized along north–south adjacent blocks at a 0.76 m alley in early June 2020 when the corns reached a V3 growth stage. Maize was grown in a split-plot design sized approximately 5.33 m in length and 0.76 m in width, which is rounded to 4 m^2^ a plot. The field was controlled from weed by a pre-plant application of herbicide atrazine and metolachlor and by hand weeding, as needed.

### 2.2. Data Acquisition

#### 2.2.1. Field Data Collection

A full suite of phenotypic metrics of hybrid corns in the center part of the field were sampled from 369 single row plots at the R6 growing stage when corns had not yet senesced and the kernel had been fully filled (Figure 1c). The in situ phenotyping process began with cutting five plants from each plot at the ground level. After removing corn ears, the fresh weight of stover comprising stalk, leaves, tassels, and husks was recorded. The phenotyping crew used a Vermeer wood chipper to shred the fresh stover, collected a subsample of stover shreds, weighted it, and put it into a tared cloth bag. The stover samples were dried in an oven at 65 °C for at least three days, and their dried weight was obtained for stover biomass. A Will mill was used to grind further the sheds to 2 mm ground powder. A combustion analysis with a Fisons EA-1108 N elemental analyzer was performed on a 100 mg portion of the powder to estimate total nitrogen concentration. The corn ears were oven-dried to a dryness of below 10% moisture at 37 °C for about one week, after which, the kernels were shelled and weighed separately from the cobs. The kernel composition and actual moisture content was immediately measured with a near-infrared (NIR) spectroscopy Perten DA7200 analyzer (Perten Instruments, Springfield, IL, USA). The actual moisture value was reported at around 8% in ambient storage conditions and was used to correct the grain yield to a dry basis. A summarized description and calculated formula of each metric can be found in Table 1.

#### 2.2.2. UAV Data Acquisition

An aerial data collection campaign was conducted on 28 August 2020 over the study field to obtain a full set of remote sensing data (Figure 1b). The data collection date corresponded to the R5 growing stage when corns had reached physiological maturity and the kernel had been denting near their crowns. We deployed a swarm of the DJI Matrice 600 (M600) Pro hexacopter (DJI Technology Co. Ltd., Shenzhen, China) carrying various types of aerial sensors outlined in Table 2. The first UAV platform was integrated with a Headwall Photonics Nano-Hyperspec sensor (Headwall Photonics Inc., Fitchburg, MA, USA), FLIR Vue Pro R 640 (FLIR Systems, Wilsonville, OR, USA) thermal sensor, and Applanix APX-15 (Applanix Corporation, OR, Canada) global positioning system (GPS)/inertial measurement unit (IMU). The stability of the three equipment elements was warranted by a DJI Ronin MX three-axis gimbal. The second platform was hard-attached with a Velodyne HDL-32 (Phoenix LiDAR Systems, Los Angeles, CA, USA) LiDAR sensor and a Sony A7R II (Sony Corporation, Tokyo, Japan) RGB camera. It should be noted that the LiDAR sensor operates at a wavelength of 905 nm, categorized as the class 1 laser that is human-eye safe and sensitive to the same types of canopy elements. The third platform consisted of an ICI 8640 P-series (Infrared Cameras Inc., Beaumont, TX, USA) thermal camera, Sony (Sony Corporation, Japan) RGB RX10 camera, and a Micasense Altum (Micasense In., Seattle, WA, USA) multispectral camera. A Gremsy T3 (Gremsy, HCMC, Vietnam) gimbal was connected to the UAV system to frame the ICI 8640 thermal (Infrared Cameras Inc., Beaumont, TX, USA) and RGB RX10 camera (Sony Corporation, Tokyo, Japan) and adjust movements thereof, while the Micasense Altum was individually held by a custom payload tray 3D-printed using ABS plastic filament. Specifications of sensors will be discussed in the section UAV data preprocessing. In addition, each M600 Pro was equipped with a DJI 3A Pro Flight Controller (DJI Corporation, Shenzhen, China), inertial measurement unit (IMU), and real-time kinematics (RTK) Global Navigation Satellite System (GNSS) receivers, which offer a positional accuracy of 2 to 3 cm as claimed by the manufacturer.

Prior to flights, a calibration tarp with a known dimension at 3 × 3 m and three reflective panels at 56, 30, and 11% reflectance was placed within the data collection window under a UAV flight swath to be imaged for correcting geometry and reflectance of the hyperspectral cubes. Identifiable ground control points (GCPs) painted with black and white were distributed evenly at the field’s corners and alleys to act as reference points for georeferencing multiple datasets. All UAV in-flight deployments were programmed with pre-set parameters based on the collecting specifications of a designated sensor to automatically operate and collect remotely sensed data without the pilot’s involvement. The flight mission for the hyperspectral system was planned by using UgCS v.4.1 (SPH Engineering SIA, Latvia) software. In exchange for 3 cm Ground Sampling Distance (GSD) (i.e., the projected pixel size on the ground) and with the sensor lens settings, the photogrammetry tool of the software determined the average flight attitude at 48 m. We set a 40% side overlap between flight swaths for ortho-mosaicking multiple cubes. Owing to the line scanning mechanism, it is not necessary to have high forward overlap; instead, we took the minimum value of 1% and set the frame per cubes at 10,000, which is equivalent to the maximum 640 × 10,000 pixels for each raw cube. In addition, we created an area of interest (AOI) that determines the field data collection window, and whenever the UAV enters the AOI, the GPS recognizes and triggers the sensor to start capturing data or to stop if exiting the AOI. The optimal flight speed was determined at 3 m/s, which is an output of the illumination intensity, the integration time, the focal length of the sensor lens, and the preset flight attitude. A dark reference of 1000 frames per 1 cube, which will be used for radiometric calibration, was snapped with the lens cap covering on the sensor. 

Similarly, the flight mission designed for the hyperspectral system above was reused for the ICI thermal and multispectral data collection system except upscaling the forward overlap to 40% between captures. For the LiDAR data collection mission, we designed the flight paths by using Phoenix LiDAR FlightPlanner (Phoenix LiDAR Systems, Los Angeles, CA, USA) software which is proprietarily developed by the vendor. This is among only a few kinds of flight planning software that can harmoniously accommodate flight parameters for both photogrammetry (image-based) and LiDAR specifications. The vendor reported the locational accuracy (*RMSE*) of a point at a range of 3.5–5.5 cm within a 50 m flying height, and the point density, which was of our most interest, was jointly influenced by flight altitude, forward velocity (speed), and lateral (side) overlap. The LiDAR point density was estimated at 1600 points/m^2^ on average from the software after considering a LiDAR field of view at 90°, a flying altitude at 50 m, a speed of 3 m/s, and a side overlap of 70%. It is recommended for mapping mission types to design the last flight path perpendicular to the along-track flight paths, thereby enhancing point cloud co-registration [53]. The GSD estimate of the RGB camera paired with the LiDAR sensor was less than 1 cm. During point cloud colorization processing later, the point clouds can be overlaid with the RGB color information from this camera.

### 2.3. Post-Collection Hyperspectral Imagery Processing

The Headwall Nano-Hyperspec is a push-broom scanner that collects reflectance through an image split perpendicular to the flight direction. The image split is a linear array of pixels (640 spatial pixels for the sensor) with each pixel containing full spectral wavelengths, and the number of image slits increases as the UAV motion occurs. The sensor has a 12 mm lens and a horizontal field of view (FOV) of 21.1°, which gathers radiometric data in the 400–1000 nm visual and near-infrared (VNIR) region across 270 bands at a sampling interval of 2.2 nm and a FWHM of 6 nm. In addition to three GNSS antennas mounted on the upper of the UAV, there is one antenna for high-performance GPS/IMU APX-15 paired with the hyperspectral camera to monitor roll, pitch, and yaw motions. The GPS/IMU was run through a post-processing kinematics (PPK) program to improve the data quality. The accuracy of the inertial measurement unit (IMU) data from the PPK is ± 0.025° in roll and pitch, and 0.08° in yaw or heading. The total payload of the M600 was 3.65 kg, which constrains the flight time to approximately 20 min. 

Push-broom sensors are known with hardware-induced spatial noise across-track and along-track. The across-track noise or vertical striping is small differences among 640 pixels in an individual linear array caused by collecting data simultaneously and independently. The along-track noise is differences among linear arrays in each hyperspectral cube due to temporal variations when collecting sequentially [54]. Spatial pixel measurements should be homogeneous for the same feature, and temporal variations between the first array and last array should be minimal to affect the signal significantly. To minimize the noise, we conducted the flights at noon under minimal cloud conditions. Further, [55] indicated that if the UAV flies within 30 min, the variation increment is insignificant at less than 2% across spatial pixels and spectral bands.

A series of steps were carried out to preprocess hyperspectral cubes, including radiometric calibration, ortho-rectification (i.e., geometric correction), and ortho-mosaicking. Due to the proximity of UAV data collection to the ground, the atmospheric correction was assumed to be far less influenced by atmospheric effects [56]. Assisted by Headwall SpectralView software, radiometric calibration was first performed to convert raw data in 12-bit digital number (DN) format to radiance values. The cube of 1000 frames as a dark reference collected prior to the flight was subtracted from the raw DN imagery, since they are a residual current, or more precisely, randomly generated electrons, flowing through the photon-sensible lens [55]. We then converted the at-sensor radiance to the at-surface reflectance that is the standard unit for a comparison of different datasets collected from multiple areas and multiple times. An empirical line method (ELM) was performed on all imagery cubes based on the near-Lambertian tarp with three known reflectance values of 56, 32, and 11%. The orthorectification step is required to geometrically correct data cubes by using their frame indices and associated GPS timestamps obtained from the high-performance Applanix APX-15 system. The GPS time is used to look up and interpolate to the system motions (roll, pitch, yaw, latitude, longitude, flight altitude, and digital elevation model (DEM)) at the time the frame was taken. The motion offsets were parameterized via PostPac UAV 8.2.1 (Applanix Corporation, Richmond Hill, ON, Canada) to generate the post-processed smoothed best estimate of trajectory (SBET) file. SpectralView software used this enhanced GPS to ortho-rectify each pixel frame by replacing them where they were at the time of the flight (the accuracy depends on the enhanced GPS claimed by Applanix). All the radiometrically and geometrically corrected data cubes were stitched together to create one single orthoimage of the field, which is known as ortho-mosaicking.

### 2.4. Post-Collection LiDAR Point Cloud Processing

During LiDAR field scanning, the Real-Time Kinematic (RTK) operation mode was initiated relying on an on-board GPS receiver (tracking x, y, z point coordinates) and IMU (tracking the sensor motions and orientation). A linear quadratic estimation (LQE) operates to integrate GPS and IMU signals to produce a statistically optimal estimate of the sensor’s position at any point in time. This mode allows generation of the LiDAR data in the point-cloud format and visualization of them in real-time in Phoenix SpatialExplorer software. With RTK, the data can be derived in centimeter-level precision, and thus, Post-Processing Kinematic (PPK) is necessary to enhance the data precision. We deployed the PPK on a web-based LiDARMill version 2 (Phoenix LiDAR Systems, Los Angeles, CA, USA), which consists of a sequence of 2 pipelines: NavLab and Spatial Fuser. The NavLab pipeline requires input data from the onboard GNSS/IMU and the base station to correct the flight trajectory in forward and reverse directions several times using Loosely Coupled (LC) and Tightly Coupled (TC) solutions [57]. LC is a naïve computation to fuse GNSS-derived position and velocity with IMU, which is infeasible with fewer than four satellites’ signals or in blocked areas, while TC overcomes the shortfall of interrupted signals by directly using GNSS static raw observations [58]. The Spatial Fuser pipeline in LiDARMill fuses the corrected NavLab trajectory data with the raw LiDAR data to generate a point cloud and further colorize the point cloud if the RGB images are inputted. LiDARMill eventually delivers a classified (ground/non-ground) point cloud and its attributes such as intensity, RGB values, number of returns in the LAS (LASer) format.

The LAS file was then used to generate raster data representing canopy height and intensity. Canopy height is a normalized surface that is the difference between the digital surface model (DSM) and digital terrain model (DTM). We created the DSM raster by first filtering the points to only non-ground and removing outlier points that lie alone in low-density regions whose the nearest neighbors are too far away. We voxelized the point cloud to a bin of small cells (voxels) at a size of 3 cm that was consistent with the pixel size of the hyperspectral image. The DSM was formed from the highest elevation cells, inside of which we selected the maximum point. The creation of DTM raster began with, first, filtering ground points and then, voxelizing of the point cloud. The triangular irregular networks (TIN) method was performed to interpolate voids found on the earth’s surface. The construction of the canopy intensity raster was similar to making the DSM except for the data type as point intensity.

**Figure 2:**
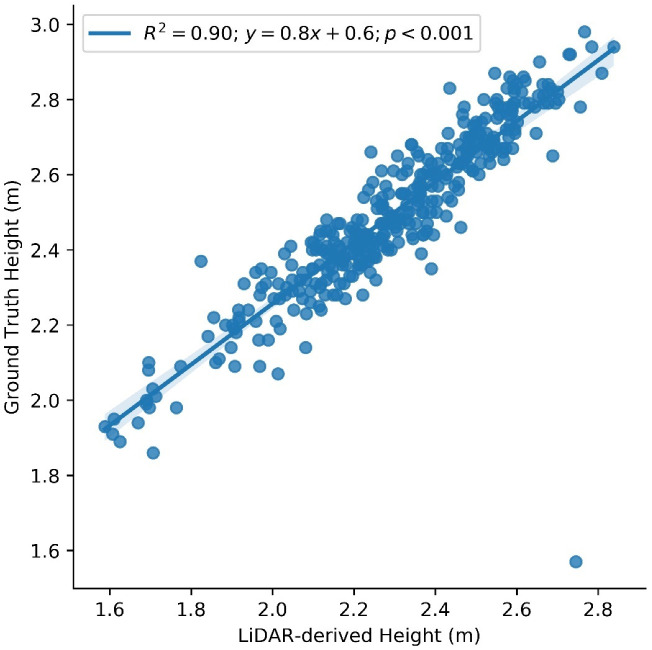
Scatter plot between LiDAR-derived (remotely captured in R5 stage) and ground truth height (measured in R6 stage) in meters.

To assure the confidence that our remote sensing data correctly captured the crop’s features, we correlated the remote sensing data, especially LiDAR data, to the actual data that were manually measured by our field management team. The ground truth height recorded the average of every three plants in the middle of each plot in the R6 stage. The remotely sensed LiDAR height was extracted from 90 percentile of the plot height to preclude aerial dust at the very top of the plot canopy. The correlation between the two showed a very strong and statistically significant degree at *R*^2^ = 0.9, *p* < 0.001 (Figure 2).

### 2.5. Post-Collection Thermal Imagery Processing

An ICI thermal sensor recorded the data in DN values in a JPG imagery format, and therefore, radiometric calibration for the thermal imagery is required to convert the at-sensor data type to a physical meaning data type at the surface–canopy temperature in Celsius degrees. This process was done in a batch through IR-Flash (Infrared Cameras Inc., Beaumont, TX, USA) software with an internally installed factory calibration file. Users are further allowed to optionally adjust environmental conditions, thermal emissivity, transmission, and ambient temperature. The converting software outputted thermal images in 32-bit TIFF format with geo-tags. The batch of radiometrically corrected images was loaded in a photogrammetric software Pix4D mapper (Pix4D SA, Prilly, Switzerland) for ortho-rectifying and mosaicking to create a single image of a captured field. A Pix4D mapper utilizes a suite of photogrammetry and computer vision techniques for extracting image key points in each image, matching the key points, stitching images together, and blending overlapping areas in the stitched ortho-mosaic.

### 2.6. Image Co-Registration

Image co-registration is the process of geometrically aligning two or more images to integrate or fuse corresponding pixels that represent the same objects or locations on the ground [59]. Although all hyperspectral images, canopy height and intensity images, and thermal images were correctly georeferenced at the same projection, they were still misaligned, typically at a centimeter level with the UAV scale. Co-registration occurred by obtaining the geometric relationship between a base image and warped images through a number of tie points. The UAV hyperspectral ortho-image served as the base image, and the LIDAR canopy height, intensity, and thermal images were warped to be closely re-aligned. A minimum of 20 tie points was manually selected, including GCP reference panels and distinct features that were evenly distributed across the field. The tie point selection was edited on the geometric correction module of ENVI 5.5 software (Harris Geospatial, Boulder, CO, USA). The software module then required users to choose warping and resampling values. A second-order polynomial was used for the warping transformation, whereas cubic convolution was used for resampling warped images, especially thermal images from 10 cm to 3 cm.

## 3. Methods

**Figure 3:**
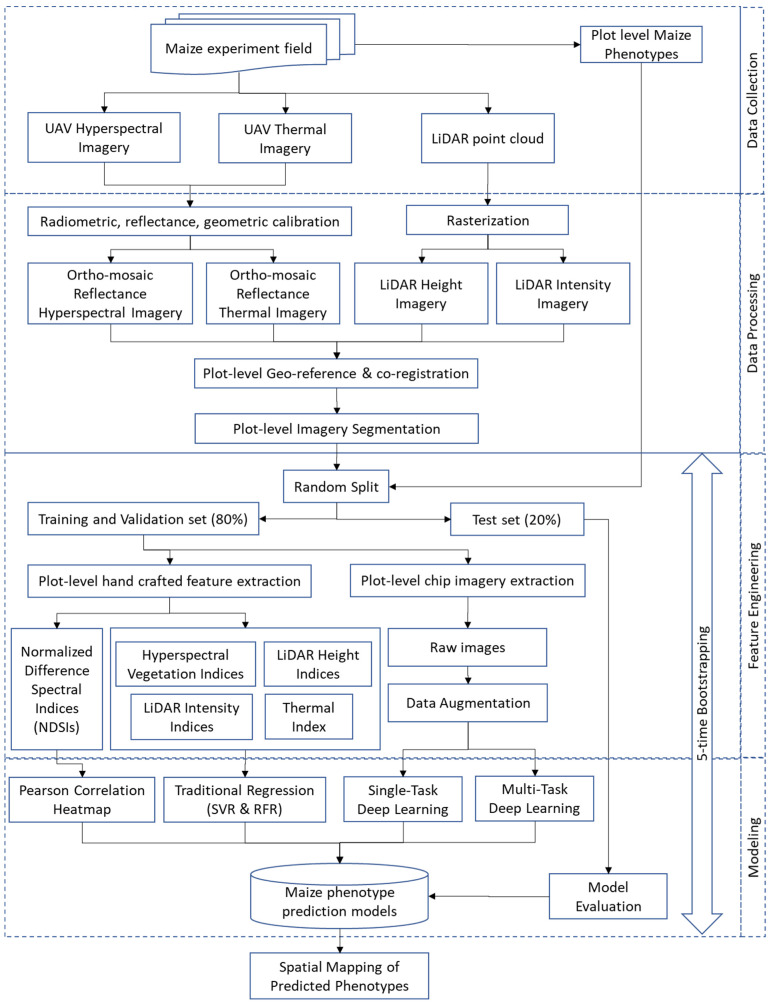
Overall workflow implemented in the study. There were four main stages: UAV data collection, remotely sensed data processing, feature engineering, and modelling. Because of a small sample size and the existence of extreme values in the data, the feature engineering and modelling phases were iterated 5 times by randomly shuffling the datasets (bootstrapping). The best predicted phenotypes were eventually plotted on spatial maps as final deliverables.

The methodology was graphically illustrated in Figure 3, an overall workflow. The methods could be partitioned into four main areas: data collection, post-collection processing, feature engineering, and modeling. The UAV aerial data collection and post-collection data processing were fully discussed in the Section 2 above. The next sections described feature engineering, both manually and automatically, and modeling methods. The predicted results were geo-located on a spatial map for a residual randomness testing (i.e., spatial autocorrelation) and eventually, for visualization.

### 3.1. Ground-Truth Data Exploration

Table 3 summarizes descriptive statistics of maize phenotypes harvested at the end of the growing season. It is discernible that all three phenotypes, dry grain yield (kg/ha), grain nitrogen content (kg/ha), and plant nitrogen content (kg/ha), rendered a high coefficient of variation (46, 53.9, and 51,7%). Rather than a bell-shaped curve, the data distribution exhibited a bimodal curve, which is a direct response to the nitrogen experiment. The low values were sampled from low N plots (i.e., no supplemental nitrogen), and high values corresponded to high N plots (i.e., 225 kg/ha nitrogen fertilizer treatment). The other five target variables had various levels of extreme instances, which skewed their distribution and would possibly negate predicting performance. This matter becomes the most obvious in a left-skewed distribution of grain density, in addition to a very narrow data range (1.02–1.35 units) and small coefficient of variation (3%).

**Figure 4:**
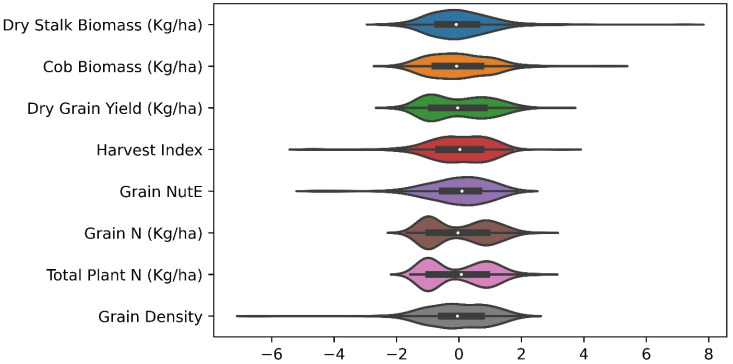
Standardized form of ground-truth data of eight maize phenotypes collected during the growing season. The means of all phenotypes after rescaling the distribution values were 0 and standard deviations were 1. Dry grain yield, grain nitrogen content, total plant nitrogen content displayed a binomial data distribution with no extreme instances. The other distributions looked normal but contained extreme values in their datasets.

It is necessary to understand the correlation degree for each pair of phenotypes collected (scatter plot matrices in Appendix A). The correlation pairs of dry stalk biomass, cob biomass, dry grain yield, grain nitrogen content, and plant nitrogen content presented a linear positive relationship, interpreted as, for example, the higher the stalk biomass is, the more likely the cob has a higher biomass. It becomes plainly visible between grain nitrogen and plant nitrogen content. Data points of harvest index and grain density were found to be dispersed when cross-plotted with other phenotypes. It is worth mentioning that grain nitrogen utilization efficiency persistently segmented its data into two high- and low-value clusters, which correspond to high and low nitrogen treatment. Given such negated features of the phenotype variables, it is advisable to implement transforming and standardizing them prior to a formal process. It is also important to project all values into a comparable scale for later multi-task deep learning and the loss function of the model, which was discussed in detail in the data transformation section below. Figure 4 therefore showed the standardized form of ground-truth data of eight maize phenotypes after rescaling the distribution values.

### 3.2. Plot-Level Chip Image Segmentation and Feature Scaling

**Figure 5:**
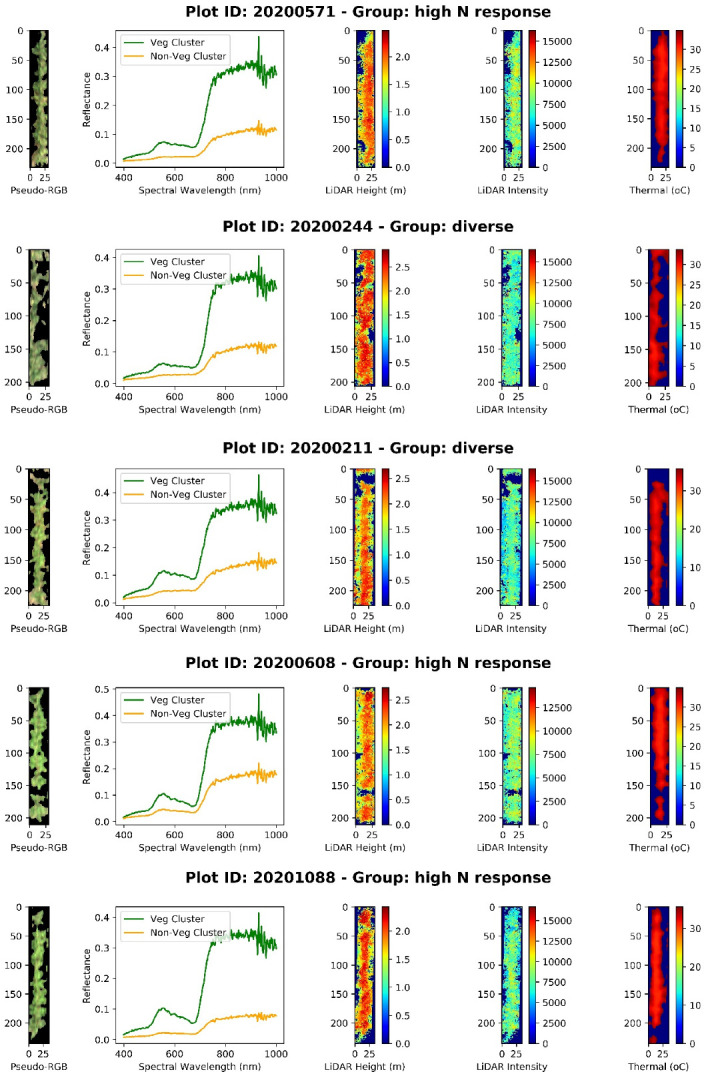
The segmentation results of 5 random plots using *k*-means clustering to extract pure vegetation pixels (Areas of interest, AOIs) from non-vegetation pixels (soil, crop residuals, heavy shadow, etc.). The segmentation was done on weed-free sample plots instead of the ortho-mosaic image of the entire field that possibly contained weeds. The hyperspectral profiles of pure vegetation were again verified and affirmed with LiDAR height and intensity, and thermal.

The remotely sensed data in this study consisted of hyperspectral imagery, LiDAR point cloud, and thermal imagery. Plot level chip images of each data type extracted from the ortho-mosaic raster of the whole field using vector data of plot boundaries (Figure 1d). It would matter if the plot images contain not only areas of interest (AOIs) maize pixels but also a various degree of confounding objects such as soil, residuals, shadow, etc. The non-AOIs were also affirmed by visually crosschecking in all data modalities: for example, the non-AOI shadow pixels valued at 0 in the LiDAR height and intensity or thermal chip images. To alleviate the matter and elevate prediction accuracy, it is suggested to segment out these confounding pixels. As the segmentation task runs on the entire spectrum, a simple method such as threshold proves to be insufficient to detect AOIs, especially mixed pixels and shaded canopy region, as studied by [60]. Instead, a unsupervised *k*-means++ clustering [61] was chosen as it receives the most popularity in both academia and industry because of an easy implementation and a high computational efficiency even with high dimensional data [62]. Only one drawback of the *k*-means clustering refers to the arbitrary user input for an a priori *k* number of clusters. This was not our case when each plot essentially had two classes, vegetation and non-vegetation (Figure 5). The multimodal data also existed in different measurement scales: reflectance spectra were in the range of 0 and 1, LiDAR-derived canopy height in meters, LiDAR intensity unitless, and thermal in Celsius degrees. After removing non-AOIs pixels, standardization (a.k.a. feature scaling) is a crucial step to ensure all features on the same scale before feeding them into machine learning algorithms.

### 3.3. An Extended Normalized Difference Spectral Indices (NDSIs) as a Simple Fusion

Normalized difference spectral indices (*NDSI*) involves statistically normalizing two spectral bands in hyperspectral data that could be best sensitive to plant’s phenotypes. Recent studies demonstrated that a *NDSI* correlation map is useful for identifying the optimal normalized indices to predict biochemical, biophysical, and structural properties [63,64]. We extended the conventional *NDSI* and applied it to other types of our data including LiDAR canopy height band, LiDAR intensity band, and thermal band. The extended *NDSI* served as a naïve fusion method to combine and normalize not only two spectral bands but also each spectral band with LiDAR height, intensity, and thermal data by following Equation (1):

(1) $$NDSI\;\left(i,j\right)=\frac{M_{i}-M_{j}}{M_{i}+M_{j}}$$

where $M_{i},\;M_{j}\;$are plot-wise mean values of raster band *i* and raster band *j*. All possible combinations (*i*, *j*) of 269 available spectral bands, 1 canopy height band, 1 canopy intensity band, and 1 thermal band were used for *NDSI* calculations for each phenotypic trait. 

### 3.4. Feature Engineering and Traditional Machine Learning

Feature engineering is an essential step that applies hardcoded transformations to raw data, which makes the data more amenable to machine learning algorithms. It especially matters if the input data are high-dimensional, such as hyperspectral images, wherein the number of features is substantially higher than the number of samples. If not being properly engineered, unrelated pixels in the spatial domain and multicollinear spectral bands in the spectral domain could possibly add more noise and diminish the model generalization. Establishing vegetation indices (VIs) from high-dimensional data is a common technique in vegetation remote sensing research. A set of 34 VIs representing maize phenotypic expressions (biochemical, biophysical, physiological, etc.) was extracted from plot-wise hyperspectral cubes, conventionally used in previous studies [18,25]. A similar index formation on LiDAR and thermal data [65,66] generated 30 VIs from the height statistics, 30 VIs from the intensity characteristics, and 1 thermal-derived VI. Table 4 enumerated all VIs notions and their meanings.

A machine learning pipeline was then constructed with two regressors: Support vector machine for regression (SVR) [67] and Random forest regression (RFR) [68]. Both are versatile and widely accepted methods in the vegetation remote-sensing community. SVR gives a flexibility to define how much error is acceptable through finding an optimal error tube (the separating hyperplane or decision boundary in the classification problem). To achieve a small error, we experimented on the SVR with a hyperparameter GridSearch library. The first hyperparameter *C* controls the width of the margin, and when C is large, the SVR tends to be overfitting, while when C is small, the SVR tends to be underfitting. Second, the kernel function, which creates nonlinear combinations of the original features to project them onto a higher-dimensional space via a mapping function, where the new transformed data become linear. γ is the third hyperparameter to be optimized, controlling the influence of the similarity distance. The smaller the values of γ, the larger the similarity radius, whereas with high values of  γ, the data examples must be closer to be affected. 

RFR is ensemble learning that combines several base learners (i.e., decision trees) into a meta-learner in order to achieve a better performance than each individual model alone. A similar hyperparameter tuning was done in a 5-fold inner cross-validation, as [69] recommended. The first hyperparameter was the number of decision trees (*k*). With fewer *k*, the model variance tends to increase and the meta-learner is prone to overfitting, whereas the model bias remains constant. The next hyperparameters were the maximum depth that a tree can grow and the minimum number of samples at the leaf nodes. RFR also measures the feature importance of a feature to the predicting power toward the target (i.e., maize traits). It is also known as the mean decrease impurity (MDI) and will be used to assess the importance of each data modality towards the model’s predictive power later in Section 5 discussion.

### 3.5. Multimodal Fusion and Multi-Task Deep Learning

#### 3.5.1. Deep Learning and the Need for Data Augmentation

Deep learning prediction performance could generally achieve its potential when training on a sufficiently large dataset. This is valid partly due to its nature and capability for searching relevant and salient features in the training data without any need for manual feature engineering, which can only be done on the availability of a large amount of data. Many have shown that data augmentation improves the generalization performance and reduces overfitting on a small dataset [97,98]. We attained more data samples by iterating random cropping on each plot boundary via a restricted ‘field of view’ (FOV). The FOV was the actual plot size at 5.33 m × 0.76 m, equivalently, 176 pixels in length and 25 pixels in width at 3 cm GSD, whereas the plot boundaries were fairly larger because the mature plants traverse over the allies. The spatial 176 × 25 window randomly and iteratively sliced 20 times on each plot to cover enough every corner of a plot but with not too much overlapping among cropped images in the dataset. For each cropping iteration, a random number generator was set the same across hyperspectral, LiDAR-derived, thermal images to ensure the sliding window was cropping the same region within each plot. The augmentation procedure was solely applied on the training set.

#### 3.5.2. Convolutional Neural Network for Imagery Representation Learning

The convolutional neural network (CNN) has gained huge popularity in the application of deep learning in the last decade due to its efficient and robust performance toward learning salient representations or relevant features of the imagery data format [62]. This study orchestrates a stack of 3D convolutional layers that can automate extracting jointly spatial and spectral representations of a 3D hyperspectral cube, relying on a hypothesis that crops exhibit their properties in both spatial and spectral domains. Particularly, we assembled four 3D convolutional layers equipped with a kernel size of 3 × 3 × 7 (3 × 3 in spatial dimension and 7 in spectral dimension) and stride of 1 pixel at a time. The number of convolutional filters started with 8 at the first layer, raising a power of 2 to 16, 32, and 64 filters. Kernel weights of each convolutional layer were initialized by sampling from Glorot uniform distribution [99]. The kernel bias was typically configured to initialize with 0. Rectified linear unit (ReLU) [100] served as activation functions due to its widespread popularity in tackling the vanishing gradient problem (gradient terms are close to or equal 0) as a network adds more layers and becomes deeper. Reducing the tensor volume by subsampling layers is a recommended practice. We experimented on two pooling forms, max pooling and mean pooling, and found that 3D max pooling layers with a size of 2 × 2 × 6 max pooling worked better because features tend to encode the spatial presence of some pattern over the different tiles of the feature map, and obtaining the maximal presence of different features became more informative. The second advantage of max pooling refers to a local invariance that means small changes in a local neighborhood do not change the result of max pooling. Similar to the 3D convolutional for volumetric learning in hyperspectral imagery, a 2D convolutional version was constructed in two separate network streams for LiDAR-derived and thermal imagery learning. 

#### 3.5.3. Multimodal Fusion and Multi-Task Prediction Block

**Figure 6:**
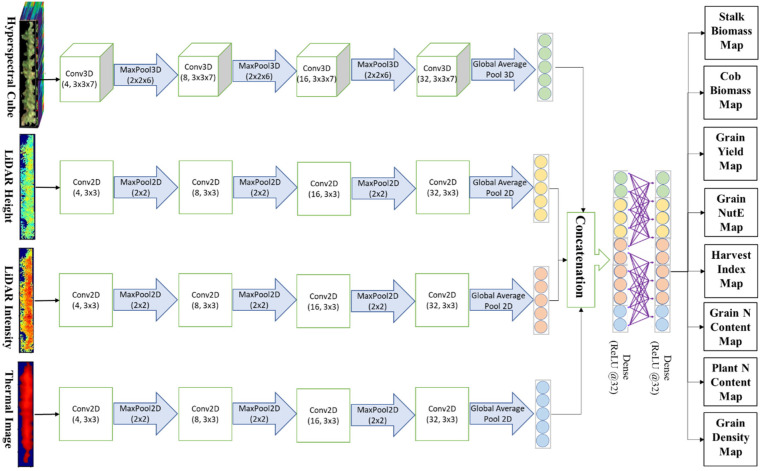
Multimodal fusion and multi-task deep learning scheme simultaneously predicting all of the maize phenotypes. Each stream of convolutional layers automatically processed a different data modality prior to being fused and fetched to multi-task regressors.

Each of the three convolutional network streams ended up with 64 feature maps of different tensor shapes that were then funneled to global average pooling layers. This helped reduce trainable parameters and simplify the model capacity, thereby minimizing the risk of overfitting. At the fusion node, we fused each of the 64 features together. Lastly, a prediction block consisted of fully connected layers carrying 32 neuron units and ReLU activation to map convolutional features to the output targets. Inserted between fully connected layers was a dropout regularization technique [101] that involves the removal of randomly selected neurons from the network’s hidden layers in each round of training. By a random dropout, the model does not memorize or become over-reliant on certain features of the data to reduce overfitting and generate a good prediction. The whole block of multimodal fusion and multi-task deep learning was graphically illustrated in Figure 6.

#### 3.5.4. Loss Function

Selection of the proper loss function is critical for training an accurate model as it measures how well the model did at predicting the outcome. Two common loss functions for a regression modeling are Mean Squared Error (MSE) and Mean Absolute Error (*MAE*), and each has certain properties. If outliers are present, the quadratic function of MSE weights more largely on anomalous errors from outliers and significantly magnifies the errors. *MAE*, however, behaves opposite to MSE, as it applies the absolute value to the difference between the predictions and ground truth, thereby averaging it out across the entire dataset. This property makes *MAE* ineffective in caring about outlier predictions as the huge errors coming from the outliers end up being weighted the exact same as lower errors. The fact is that extreme cases usually occur in plant phenotyping expressions due to mutual interactions between internal and external variables such as genotypes and environmental conditions. Huber loss function [102] offers the best of both worlds by harmonizing MSE and *MAE* using the following piecewise Equation (2):

(2) $$L_{\delta }\left(y,f\left(x\right)\right)=\left\{\begin{array}{c} \frac{1}{2}{\left(y-f\left(x\right)\right)}^{2}\;\;\;\;for\;\left|y-f\left(x\right)\right|\;\leq \;\delta \\ \delta \left|y-f\left(x\right)\right|-\frac{1}{2}\delta^{2}\;\;\;for\;\left|y-f\left(x\right)\right|>\delta \end{array}\;\right.$$

where y is the actual (true) value of the target data point, $f\left(x\right)$ is the predicted value of the data point. δ defines a threshold where the Huber loss function transitions from quadratic to linear. δ is a hyperparameter to be tuned in which the Huber loss approaches *MAE* when δ is asymptotic to 0 and MSE when δ becomes larger.

The deep learning architecture was implemented using TensorFlow (tensorflow.org) and Keras (keras.io) Python libraries. The splitting ratio of 60–20–20% was used in training, validation, and test samples. To assist the model to find the global minima and achieve the lowest loss, we adopted several widely recommended techniques such as the Adam (adaptive moment estimation) optimizer with a scheduled learning rate (started at 0.001 and exponentially decreased every 5 epochs).

### 3.6. Model Evaluation and Performance Metrics

To evaluate the performance across prediction models, the coefficients of determination (*R*^2^), root mean square error (*RMSE*), and mean absolute errors (*MAE*) were computed and contrasted, which can be expressed as follows:

$$R^{2}=1-\frac{\sum_{i=1}^{n}{\left({\hat{y}}_{i}-y_{i}\right)}^{2}}{\sum_{i=1}^{n}{\left(y_{i}-{\bar{y}}_{i}\right)}^{2}}$$

$$RMSE=\sqrt{\frac{\sum_{i=1}^{n}{\left({\hat{y}}_{i}-y_{i}\right)}^{2}}{n-1}}$$

$$MAE=\frac{1}{n}\overset{n}{\underset{j=1}{\sum }}\left|y_{i}-{\hat{y}}_{i}\right|$$

where ${\hat{y}}_{i}$ and $y_{i}\;$ are the measured and the predicted values, respectively, $\bar{y}\;$ is the mean of the measured value, and *n* is the total number of samples in the testing set.

Further, a spatial variability of the prediction results was statistically evaluated, particularly by Global Moran’s I (GMI). The GMI measures the spatial autocorrelation contingent on the maize plot locations and the model’s regression errors over the study area [5,103]. The errors were residuals between the measured and predicted phenotypes of each maize plot. The GMI’s null hypothesis states that the phenotypes’ predicted errors are complete spatial randomness or randomly distributed.

## 4. Results

### 4.1. Results of a Naïve Fusion NDSI Method

**Figure 7:**
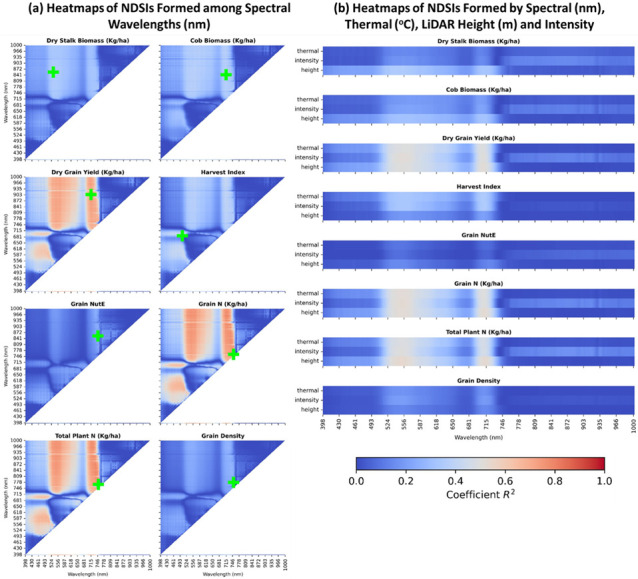
Extended *NDSI* correlation heatmaps. Each *NDSI* was established by combining and normalizing two 269 singular spectral bands (398 nm–1000 nm) (**a**), and fused features from spectra, LiDAR canopy height, LiDAR canopy intensity, and canopy thermal data (**b**). The lime-colored cross signs indicated the best *R*^2^ for each maize trait. In detail, Dry Stalk Biomass achieved a maximum *R*^2^ = 0.357 with *NDSI*_[534, 868]_. Cob Biomass optimally gained *R*^2^ = 0.384 at *NDSI*_[715, 855]_. Dry Grain Yield had the highest *R*^2^ = 0.746 at *NDSI*_[715, 917]_. Harvest Index received the highest *R*^2^ = 0.451 at *NDSI*_[504, 700]_. Grain Nitrogen Utilization Efficiency (NutE) attained *R*^2^ = 0.271 at *NDSI*_[749, 866]_. Grain Nitrogen Content (Grain N) reached *R*^2^ = 0.796 at *NDSI*_[751, 769]_. Total Plant Nitrogen Content (Total Plant N) had the peak of *R*^2^ = 0.80 at *NDSI*_[751, 778]_. Grain Density ran into *R*^2^ = 0.276 at *NDSI*_[751, 789]_.

The extended *NDSI* method was a fast and naïve approach for fusing all 269 spectral bands, LiDAR canopy height and intensity, and thermal data. Figure 7 discloses the correlation degree between the established *NDSI*s and maize phenotypic traits through *R*^2^ heatmaps with a same scale of 0–1 (dark blue to dark red). The figure glimpsed that *NDSI* heatmaps formed solely from spectra (Figure 7a) had regions having a higher degree of correlations than those in the heatmaps formed from spectra, thermal, LiDAR height, and intensity (Figure 7b). All eight highest *R*^2^ (lime cross sign) were found in Figure 5a’s heatmaps. Equivalently, dry stalk biomass received the highest *R*^2^ = 35.7% when correlated with the *NDSI*_[534, 868]_. Cob biomass correlated with the *NDSI*_[715, 855]_ at *R*^2^ = 38.4%. The *R*^2^ of dry grain yield reached up to 74.6% by the *NDSI*_[715, 917]_. Harvest index peaked at *R*^2^ = 45.1% by the *NDSI*_[504, 700]_. The correlation of grain nitrogen utilization efficiency (Grain NutE) with the *NDSI*_[749, 866]_ made the highest *R*^2^ = 27.1%. The *R*^2^ for grain nitrogen content equaled 79.6% at the *NDSI*_[751, 769]_, and the total plant nitrogen content *R*^2^ was 80% by the *NDSI*_[751, 778]_. The *R*^2^ of grain density achieved 27.6% as the highest value at the *NDSI*_[751, 789]_.

The common theme running through all heatmaps was the contributory significance of green bands (530–560 nm) and red-edge bands (700–720 nm) in the spectra. Those bands pairing with NIR bands (750–1000 nm) to create *NDSI*s correlated best with dry grain yield, grain nitrogen content, and total plant nitrogen content. It is noted that the simple data fusion *NDSI* by combining and normalizing spectral bands, LiDAR canopy height, LiDAR canopy intensity, and thermal features correlated with eight maize phenotypic traits at a minimal degree. This clues a necessity for a complication of extracting explanatory features from each data source and fusing them effectively.

### 4.2. Machine Learning and Deep Learning Performance on Multisensory Fused Data

**Figure 8:**
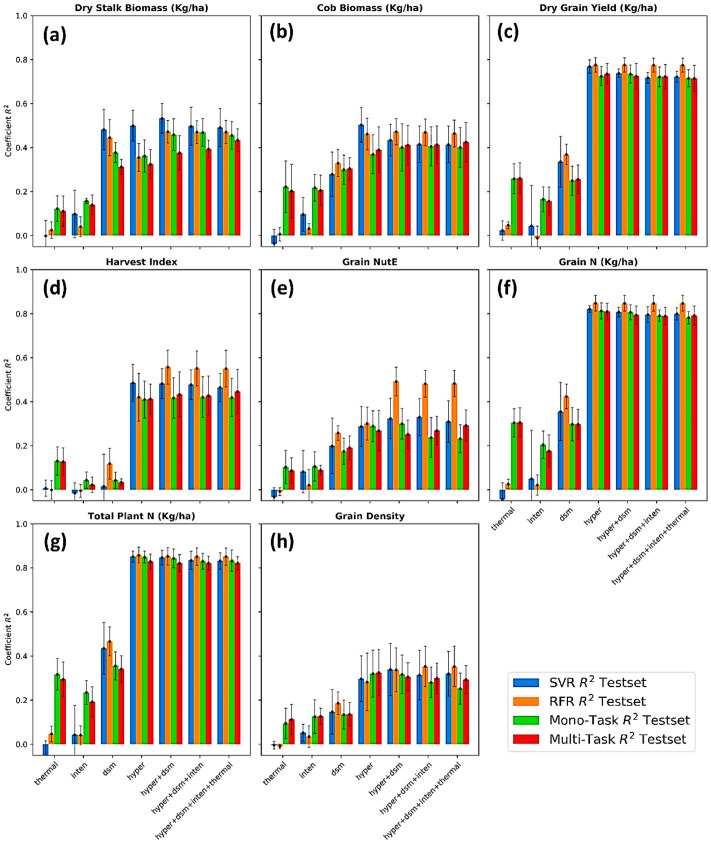
Prediction performance of eight maize phenotypes (**a**–**h**) represented by *R*^2^ across different feature types and regressors. Feature types included thermal = canopy thermal, inten = LiDAR canopy intensity, dsm = LiDAR canopy height, hyper = hyperspectral images. Feature fusions included hyper + dsm = a fusion of hyperspectral and LiDAR canopy height, hyper + dsm + inten = a fusion of hyperspectral, LiDAR canopy height, and LiDAR canopy intensity, and hyper + dsm + thermal = a fusion of hyperspectral, LiDAR canopy height, LiDAR canopy intensity, and thermal.

Figure 8 demonstrates the mean and standard deviation of coefficient *R*^2^ performed on a 5-time bootstrap using four different regressors and a variety of multi-sensory data fusions. The following common points can be noticed from Figure 6, and more details (*R*^2^, *RMSE*, *MAE* of both train and test sets) can be accessed in Appendix B. First, the prediction success highly varied from phenotype to phenotype, possibly dividing into a limited (*R*^2^ < 0.5), moderate (0.5 < *R*^2^ < 0.8), and high level (*R*^2^ > 0.8). Predictions of dry grain yield (*R*^2^ = 0.78), grain nitrogen content (*R*^2^ = 0.85), and total plant nitrogen content (*R*^2^ = 0.85) were reported as the highest degree of success. Although different studies employed different methods and data available, this study’s results were somewhat better to recent studies of maize yield prediction (*R*^2^ varied 0.3–0.8 depending on growing stages) [104,105,106], total Nitrogen content (*R*^2^ = 0.76) [107].

Predicting dry stalk biomass (*R*^2^ = 0.53), cob biomass (*R*^2^ = 0.47), harvest index (*R*^2^ = 0.56), and grain NutE (*R*^2^ = 0.49) came in second at a moderate success. There is no direct comparison, but recent studies of maize above ground biomass (AGB) predicted more accurately than our results at *R*^2^ = 0.86 [108] and *R*^2^ = 0.87 [109]. Prediction results of grain density (*R*^2^ = 0.34) showed a limited success. The varying prediction success can also be seen through the error bars of each model; for example, models predicting dry stalk biomass (Figure 8c) had smaller deviations when shuffling the dataset, while the deviation of model predicting grain density was considerably wider. This proved that grain NutE and grain density contained extreme values in the dataset, and when being shuffled and randomly split, the train sets and test sets did not warrant an equivalence. The substantial disparity between the *MAE* and *RMSE* (Appendix B) also suggested the existence of a very wide and inconsistent data range of the two maize traits. This matter could be typically dissolved by collecting more samples, which is recommended to future studies.

Second, the prediction success highly varied from data type to data type. Models deploying with data types of either hyperspectral singularity or hyperspectral fusion can produce a sustainably better estimate for maize phenotypes in comparison to models using thermal and LiDAR canopy intensity. On the other hand, models without the inclusion of hyperspectral data, which are thermal, LiDAR intensity, and LiDAR height, presented a limited success in predicting all maize traits. The variation of those models on shuffled data being represented by the error bars in Figure 6a–h was also larger than the variation of models with the presence of hyperspectral features.

Third, machine learning and multi-task deep learning methods performed the regression comparably with a little disparity of *R*^2^, *MAE*, and *RMSE*. The RFR regressor occasionally proved to be a slightly more accurate estimation (higher *R*^2^, Figure 8d,e), but the multi-task learning method occurred as more stable by a narrower deviation (error bars, Figure 8f,g). Noticeably, if considering models with only thermal and LiDAR intensity for all eight maize traits (Figure 8a–h), traditional machine learning can do the task minimally, while a much higher prediction accuracy was observed in deep learning regressors. This reflected that the SVR and RFR heavily relied on handcrafted features in which only a single thermal index was manually extracted and deployed, while the deep learning regressors perhaps grasped many informative features from the raw thermal images.

### 4.3. Spatial Distribution Maps of Predicted Results

**Figure 9:**
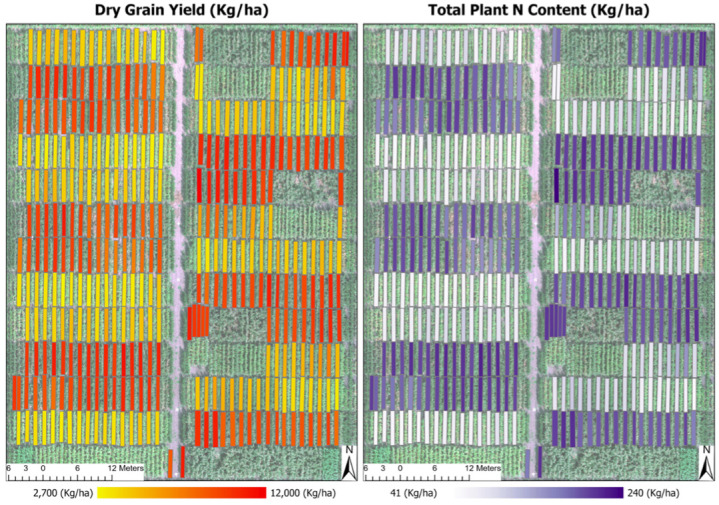
Spatial distribution maps of predicted dry grain yield (kg/ha) and total plant nitrogen content (kg/ha) which were the results of the multimodal and multi-task deep learning model.

Figure 9 projected the predicted values of dry grain yield and total plant nitrogen content at a plot level on spatial maps. The two maps were results from the multi-task learning model performing the prediction on the fusion of hyperspectral and LiDAR canopy height imagery data. It is necessary to notice that only these two results were graphically displayed on the maps due to a page limit of an article, and interested readers are encouraged to contact the authors and request a complete copy of the digital maps.

From the maps, it is visually detected that the distribution of predicted values clustered into plot blocks of low and high values of both grain yield and plant nitrogen content traits. These low- and high-value blocks were consistently aligned with the blocks annotated with the nitrogen experiment. It means that low-value plot blocks corresponded to the control blocks without nitrogen addition, whereas high-value plot blocks paired with the experiment blocks with 225 (kg/ha) nitrogen fertilizer per each plot. Further, the models returned the predicted values spanning within a narrower range of 2700 to 12,000 (kg/ha) for grain yield and 41 to 240 (kg/ha) for plant nitrogen content, compared to the actual values of 425 to 17,450 (kg/ha) and 26 to 314 (kg/ha), respectively (Table 3, statistics of ground truth data). This matter occurred due to the possibility of the Huber effect set as the loss of the models. Too extreme values in both ends were constrained by the Huber loss, as such, making the regression errors smaller.

## 5. Discussion

### 5.1. Remote Sensing Data for High-Throughput Maize Phenotyping

The results in the preceding section promoted a varying success of maize phenotype predictions with a use of multi-sensors UAV at very low altitude and high resolution. It is strongly desired to have an innovative tool for high-throughput maize phenotyping by estimating all traits at a time; however, the fact is that each crop phenotype has its own mechanism that dissimilarly reacts to the nitrogen experiment, not to mention the environmental conditions at different times in a day [110,111]. Eight maize plant trait in this study belongs to different categories: biophysical (stalk biomass, cob biomass, harvest index) biochemical (plant nitrogen content), and maize grain traits (grain yield, grain nitrogen content, and grain density).

**Figure 10:**
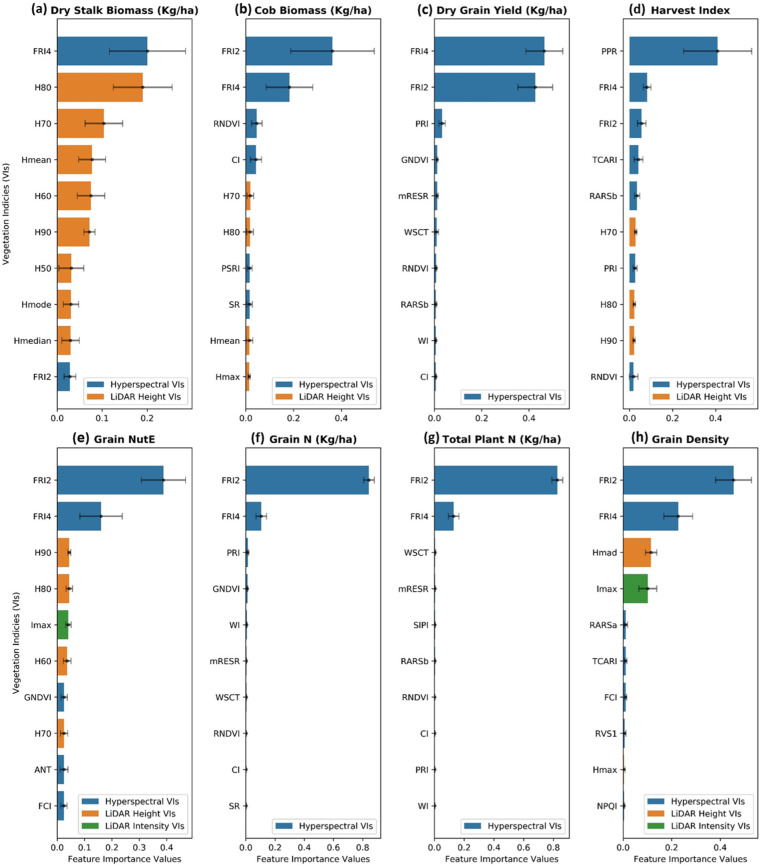
The 10 most important vegetation indices in a descending order of feature importance analysis performed by the mean decrease impurity (MDI).

The significance of optical remote sensing, especially the NIR spectra (750–1000 nm) in all eight maize estimations was demonstrated. The wavelengths most important for predictions are detailed in an ascending order: 749 nm, 751 nm, 769 nm, 778 nm, 789 nm, 855 nm, 866 nm, 869 nm, and 917 nm (Figure 7). More concretely, the mean decrease impurity (MDI) feature importance analysis (Figure 10) unfolded the two most critical VIs for predictions, namely, Fluorescence Ratio Index 2 and 4 FRI2_[750, 800]_ and FRI4_[740, 800]_ in the form of NIR wavelengths (740 nm, 750 nm, and 800 nm). It has become obvious that the near-end NIR simulates molecular motion of compounds residing in internal leaves that induces a strong reflection of downwelling radiance [112,113,114,115,116]. The NIR spectral pattern is also primarily influenced by internal scatterings in the cell structure and air-filled space, and the interaction of irradiance with starch, oil, proteins, and further compartments inside the cells, cell walls and membranes [117,118]. It is worth mentioning that the water content of leaves and plants can be characterized in the far-end wavelengths (greater than 900 nm) in the NIR region [119]. Being able to remotely sense the above-stated compositions from the aerial level greatly benefited estimating not only canopy and plant phenotypes but also grain-related traits since the elements are transported from stems to the corn ears and eventually ended up at kernels.

Stalk biomass was found to be estimated the most accurately by fusing data modalities. In addition to the valuable contribution of the NIR spectra discussed above, canopy and plant structural descriptions derived from LiDAR data such as canopy height and intensity served as critical sources of information to predict stalk biomass. More obviously, Figure 8a informed the dominance of the crop’s structural features when 8 out of 10 of the most important features were descents from the LiDAR canopy height. Many studies came to a consensus that vegetation spectra alone are insufficient to access a high accuracy of stalk biomass prediction due to a vegetation saturation effect. [112,120] explained that this effect likely occurs when the crops canopy outstretches and reaches to a 100% cover in the mid-vegetative period, while most crops’ biomass continues accumulating under the cover. In this context, the absorbed and reflected amount of downwelling radiation remains virtually unchanged, but the stalk biomass is more likely to increase, making it harder to predict. Our study reinforced that the effect was substantially lessened by taking structural descriptions such as LiDAR derivatives into the model.

### 5.2. Contribution of Different Data Modalities for Phenotyping Predictions

**Figure 11:**
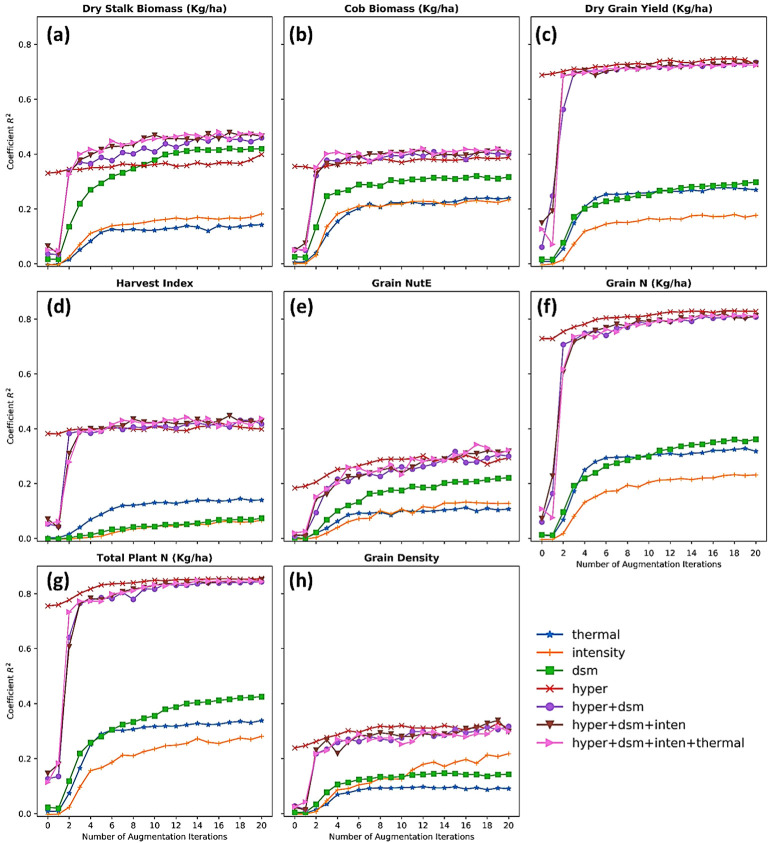
The effect of data augmentation on the prediction performance of multi-task deep learning with different data modality and fusions. Data types included thermal = canopy thermal images, inten = LiDAR canopy intensity images, dsm = LiDAR canopy height images, hyper = hyperspectral images. Feature fusions included hyper + dsm = a fusion of hyperspectral and LiDAR canopy height images, hyper + dsm + inten = a fusion of hyperspectral, LiDAR canopy height, and LiDAR canopy intensity images, and hyper + dsm + thermal = a fusion of hyperspectral, LiDAR canopy height, LiDAR canopy intensity, and thermal images.

At the time, this study utilized and encompassed all of the state-of-the-art sensors tailored to a small UAV for phenotyping scouting. In this section, the potential of each data type was explored on the basis of both individual and joint contribution toward a variety of maize phenotyping predictions. First, relying on the results from Figure 8 above, the hyperspectral data were the modality, whether existing in a form of indices or imagery or in a type of singularity or fusion, being able to substantially boost the regression performance. Further analyses, including MDI feature importance (Figure 10) and a sensitivity analysis of imagery augmentation (Figure 11), disclosed that hyperspectral data in both indices and imagery format stood up as the most contributory predictor. Many of the previous studies have acknowledged the great value and applicability of UAV-borne hyperspectral imaging (HSI) on the basis of a better performance profiling vegetation properties and respective endmembers by a contiguous spectra record and storage [16,18,60].

In spite of the proven value of the hyperspectral, there was an exception with respect to predicting maize stalk biomass when the LiDAR-derived canopy height became a more predictive power than the hyperspectral (Figure 10a and 11a). In consistency with previous studies [112,114], crop canopy height was highly correlated with biomass, and the inclusion of crop height with spectral indices improved the accuracy of the biomass prediction. In addition to the finding of LiDAR data aforementioned, this study unfolded the significance of representations of 50 and higher percentiles of the canopy height, as their indices were all displayed as the most important features, particularly for stalk biomass prediction. This implied that the upper half of the canopy structure such as stems, leave angle, tassels contains enriched materials essential for phenotyping scouting. Stalk biomass was the only trait in this study showcasing the value of LiDAR canopy height weighed over the other data types value (Figure 11a).

The third data modality investigated in this study was LiDAR-derived intensity at the canopy level. LiDAR intensity indices noticed a weak significance in predicting grain-affiliated traits (Figure 10e,h) with a standout of the Imax index (the maximum value of LiDAR canopy intensity points). The canopy roughness and scattering intensity have little quantitative meaning in remote sensing for crop monitoring; instead, LiDAR intensity could be used for qualitative analyses of the points [121]. Thermal data had the least influence on all predictive models of maize traits in this study irrespective of machine learning or deep learning regressors and of singularity data or fusion data. Graphically explained by the MDI feature importance analysis in Figure 10, the thermal index in machine learning models was completely irrelevant in maize predictions, and similarly, a negligible contribution towards the predictive power was also found in deep learning models with thermal imagery data alone (Figure 11). Previous studies showed that thermal infrared (8000–14,000 nm) remote sensing lends itself to modeling water-induced stress in crops by recognizing the plant responses, including stomatal closure, decreased transpiration, or simply leaf and canopy water content [39]. It was not the same case in this study when water was adequately supplied to all plots in the entire growing season.

Multimodal data fusion was the focal interest in this study that performed a sounder prediction than individual data modality models. It is plausible that, in as many characteristics of maize as being sensed, each of these details, itself and jointly, supplements to predicting the crop’s status. The hyperspectral provides ample information about nitrogen [122], chlorophyll and anthocyanin absorption [123], leaf cellular scattering [124], senescence [125]. LiDAR derivatives communicate plant structures and metabolism [126], whereas thermal discloses canopy temperature and water content ([127]. A 3 to 10% more accurate prediction by a fusion of multiple aerial data modalities is also found in a few articles relative to soybean yield estimation [5] and urban tree classification [128]. We examined four fusion models in this study, and the prediction performed by the fusion between hyperspectral imagery and LiDAR canopy height was most accurate.

### 5.3. Feature- and Imagery-Based Prediction Comparison

The results from Figure 6 illuminated a comparable predictive performance between feature-wise and image-wise methods with a few minor exceptions. When predicting the harvest index and grain nitrogen utilization (Figure 6d,e), the RFR performance with handcrafted features proved to be discernible relying largely on the significance of PPR_[450, 550]_ and FRI2_[750, 800]_. Comparing the CNNs deep learning assembled by multiple filters and slicing 3D kernels (3 × 3 × 6) casts doubt on whether the models experienced undesired information loss of the relationship between the above-indicated bands of 450 nm and 550 nm, and between 750 nm and 800 nm for the predictions. It was perhaps due to the 3D kernels neglecting ratios of faraway bands in the spectral dimension [129,130]. It could hardly be adjusted because the study attempted to construct a single model for multiple outputs, and future studies may want to fine tune these hyperparameters and tailor them for individual predictions of harvest index and grain nitrogen utilization.

Image-wise deep learning models’ performance discernibly beat the indices-wise machine learning models in all of the predictions if either thermal imagery and LiDAR canopy intensity imagery were inputs (Figure 8). In the same figure, given the 5-time dataset shuffling and bootstrapping, the smaller error bars of deep learning models proceeding with thermal and LiDAR canopy intensity concretely showed that image-wise models remained more stable and steadier than the indices-wise models. This result demonstrated the high quality of excellence of CNN family architectures when processing images and extracting learnable details from them [131,132]. It becomes clear that the vegetation indices can only derive a few numbers to a dozen of attributes such as the temperature mean of each whole plot, but by an operational difference, the convolutional layers can slide through all pixels of the plots’ thermal images to attain enriched and complex attractions for the predictions. Furthermore, the stability of image-wise deep learning methods could again be observed in Appendix B (a summary table of training and testing results) citing no clue of overfitting between training and testing metrics (*R*^2^, *MAE*, *RMSE*). The overfitting magnitude of indices-wise machine learning models was substantially higher, particularly when looping through shuffled datasets.

### 5.4. Mono-Task and Multi-Task Learning Comparison

In a comparison between mono-task and multi-task deep learning models, it is necessary to inform that mono-task models learned and inferred independently for each of the eight maize traits, which makes them different from multi-task models that simultaneously accomplished eight phenotypic predictions. Given the same feature fetching approach (i.e., data singularity or fusion), the results of mono-task and multi-task methods from Figure 8 were identical, but to mention that the multi-task slightly outperformed in models predicting harvest index, grain nitrogen utilization efficiency (Grain NutE), and grain density. This finding was very supportive as it was aligned with the results of [133] in that the author articulated that multi-task learning could exploit latent relatedness of crop traits during the process of optimizing weights and biases of each node in the network.

Further, the multi-task models appeared to be noticeable when inputs are an imagery fusion of hyperspectral, LiDAR, and thermal. The high performance of the multi-task even sustained throughout all fused models, while the performance of the mono-task saturated, if not slightly decreased, when adding LiDAR canopy intensity and thermal to the fused models (Figure 8). It is obvious that while LiDAR canopy intensity or thermal became noisy and corrupt data for a particular maize trait, it could be predictive data for another trait. The sharing protocol can be achieved only by the multi-task, where it leveraged the convolutional layers to extract shared information from the data fusion and allocate them to each task, if needed, to minimize the preset loss. The last and most visible advantage of the multi-task over the mono-task rested in chipping down required computational resources to a fraction and concurrently accelerating high-throughput phenotyping. Because calculating resource savings from multi-task learning was not a focus of this study, we did not document these figures, and interested readers can refer to this matter in [134,135].

### 5.5. Impacts of Data Augmentation on Deep Learning Regression

With a limited number of samples collected, it becomes difficult for any deep learning methods to be convergent during the training process and to infer a reliable result. The imbalance effect of small labeled samples and the high dimensionality of remotely sensed data is an intrinsic limitation in the remote sensing research, which is known as the Hughes phenomenon [23]. This study is not an exception when there were only 369 field plots manually measured and annotated for analyses. To address the limitation, we augmented imagery data by iterating 20 cycles of randomly slicing a spatial window over plots only on training sets. Figure 10 unveiled a boost in the *R*^2^ metric when the augmented models inferred against the test sets. With respect to the impact of the augmentation method on the models with a singularity of data types, the *R*^2^ metrics steeply ascended after a few augmentation iterations, and it continued even after 20 iterations. It bears noting that the hyperspectral images did not benefit from the augmentation cycles as much as the LiDAR canopy height when the *R*^2^ of LiDAR height-inputted models took off and overshadowed the hyperspectral model’s *R*^2^ (Figure 10a).

With respect to the impact of the augmentation on data fusion models, the results also soared up after the first three iterations and reached saturation in the 20 iterations in the models of predicting cob biomass and total plant nitrogen content. The positive impact of data augmentation was credited to slicing a fixed-size spatial window through every pixel of a plot in which details of every plant in that plot were fully captured. Adding new augmented images to deep learning models equally meant forcing the models to learn all useful details of the crop’s plots, and also meant lessening the possibility that convolutional nodes fondly remember and heavily rely on certain details, which often leads to an overfitting effect.

### 5.6. Performance of Different Methods over Space

**Figure 12:**
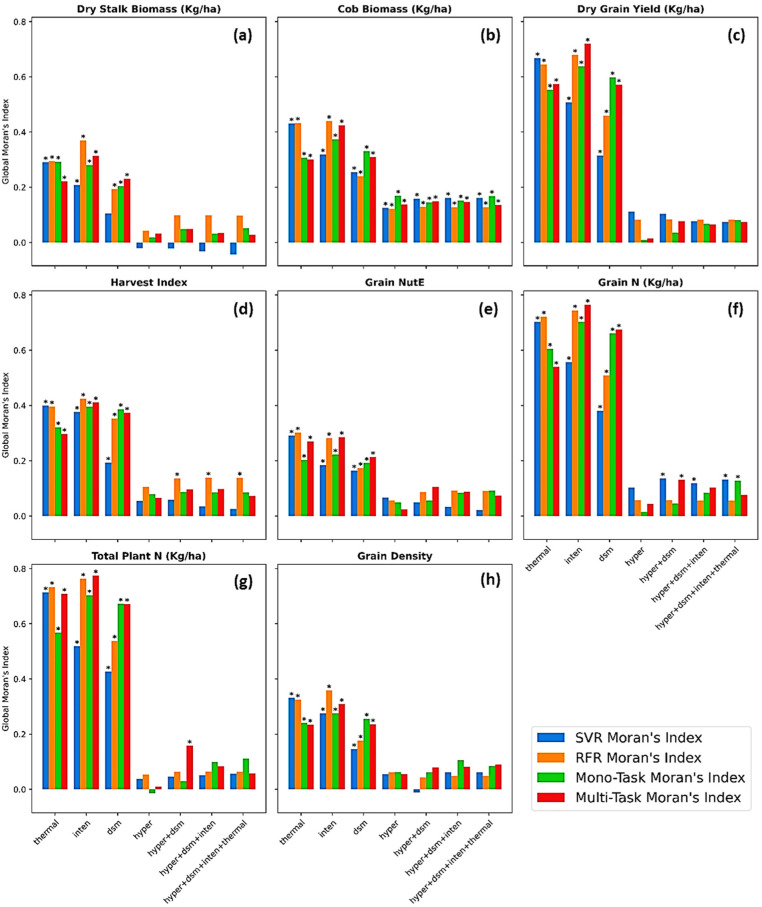
Comparison of Moran’s I values of different data types and fusions, and different regressors. The asterisk ‘*’ on the top of bars implies the Moran’s I is statistically significant at 0.001 *p*-value level. Feature types included thermal = canopy thermal, inten = LiDAR canopy intensity, dsm = LiDAR canopy height, hyper = hyperspectral images. Feature fusions included hyper + dsm = a fusion of hyperspectral and LiDAR canopy height, hyper + dsm + inten = a fusion of hyperspectral, LiDAR canopy height, and LiDAR canopy intensity, and hyper + dsm + thermal = a fusion of hyperspectral, LiDAR canopy height, LiDAR canopy intensity, and thermal.

The residuals between actual and predicted values as results of seven data sources and four different regressors were evaluated in terms of spatial randomness by GMI statistical test. Figure 12 represented Moran’s I coefficient in vertical bars colored by four methods followed by the asterisks implying a statistical significance (*p* < 0.001) of spatial auto-correlation between data points (plot prediction errors). It became obvious that regression residuals resulting from deep learning, especially multi-task learning, were insignificantly spatially correlated and remained independent from other residuals in surrounding plots. The spatial randomness was more solidly secured in deep learning models carrying hyperspectral alone and data fusion. The small and spatially random regression errors suggested an impressive prediction capability of multi-task deep learning models that could extensively apprehend complex and underlying nonlinear abstracts of imagery data of each crop plot, compared to a handcrafting establishment of vegetation indices [103]. The SVR and RFR appeared to be less reliable as their regression residuals were spatially statistically insignificant in some cases but also significant in predictions of harvest index (Figure 12d) and grain nitrogen content (Figure 12f). Additionally, the GMI test reported a significance of the regression errors from cob biomass predictions across all models and data sources (Figure 12b). The positive sign of Moran’s I coefficients noticed a clustering over the space of cob biomass prediction’s residuals. Inspecting these residuals over a map, the clusters of residuals originated from maize growing along aisles exposed the most to weather conditions. It is possible that the UAVs failed to sense certain confounding variables that could help to explain the corn cob variation, inclusive of, but not limited to, photosynthesis under the influence of sunlight intensity and metabolism with air and soil temperature progressive over time. This suggested future UAV remote sensing research to survey crops in a temporal dimension and document and incorporate field metadata into analyses.

## 6. Conclusions

With the proven success of UAV in recent digital agriculture, this study was an extended investigation of the UAV versatility for high-throughput maize phenotyping. The UAV aerial remote sensing was instrumental for scouting and estimating a full suite of eight different phenotypes in a corn field by blending geospatial and artificial intelligence (AI) competence, which is also known as GeoAI. The novelty entitling the study to be highly significant in both theoretical and practical exercises rested in the deployment of UAV airborne multisensory data fusion within a single multi-task deep learning model. Considering the results and discussions presented in the aforementioned, we concluded the following:

1. The success level of UAV multisensory data for high-throughput maize phenotyping varies from trait to trait because each trait is responsive to the experiment and environmental conditions in different mechanisms. Grain density prediction was the least successful (*R*^2^ = 0.34) in contrast with very high predictable traits: plant total nitrogen content and grain nitrogen content (*R*^2^ = 0.85). The resulting *RMSE* and *MAE* were congruent in high *R*^2^ models and became discrepant in low *R*^2^ models, which signifies extreme values in the ground dataset. Expanding observations and collecting more data are highly recommended, particularly for grain density, grain NutE, and harvest index in future research.
2. There is a varying contribution of each data modality (hyperspectral, thermal, LiDAR canopy height, LiDAR canopy intensity) individually and their fusion for phenotyping predictions. Hyperspectral data were the most primarily contributory to virtually all eight estimations, especially dry grain yield, and nitrogen content in plants and grains. LiDAR canopy height enjoyed its merit in predicting stalk biomass more accurately than any other modality. The superiority of multisensory data fusion in all phenotype predictions was evident in the study because the fusion can help to exceed limitations of single data modality, for example, the vegetation saturation effect occurring in optical remote sensing.
3. Feature- and imagery-based prediction are comparable if the latter is not superior to the former. Image-based deep learning within a framework of convolutional neural networks (CNNs) demonstrated an automation of the feature extraction, neither relying on human expertise nor being prone to human errors. This is concretely evidenced by the outperformance of image-based deep learning when thermal or LiDAR intensity data were funneled to the CNNs across maize trait predictions. The image-based deep learning remained stable as indicated by a smaller deviation through dataset shuffling.
4. Mono-task and multi-task learning are comparable if the latter is not superior to the former. Multi-task deep learning leverages latent relatedness among maize traits during optimizing cycles of weights and biases of each network node. The sharing protocol of multi-task models can reach its full potential when interacting with multisensory data fusion, which becomes multi-input multi-output models. It is also evident that executing multi-task learning models only requires a fraction of the computational resources and time needed for mono-task learning models, while accelerating high throughput phenotyping by simultaneous predictions.
5. Data augmentation for deep learning in the context of regression succeeds to elevate the intrinsic issue of a small sample size in remote sensing research (i.e., the Hughes effect). Augmented data also help to build up the rigidity and reliability of deep learning models by faster convergence and less overfitting.
6. A randomness over space of the prediction residuals from the Global Morans’ I analysis implies that there were no confounding variables implicitly veering the predictive performance of maize traits. A small and random regression error also reinforces the versatility of UAV airborne multisensory data fusion in the framework of multi-task deep learning. Cob biomass is the only trait showing a clustering pattern of prediction errors in all models, which needs to be investigated further in future research.

## Figures and Tables

**Table 1:** Descriptions of maize phenotypic traits and their measurements.

Phenotypic Traits	Unit	Calculation	Measuring Description
Cob Biomass	kg/ha	[Cob Biomass (g/plant) × Standing Plants]/Plot Size (hectare)	Average of five plants from the center of row sampled at R6 growth stage.
Dry Grain Yield	kg/ha	[Dry Grain Biomass (g/plant) × Standing Plants]/Plot Size (hectare)	Average of five corn ears from the center of row sampled at R6 growth stage. Normalized moisture content of dry grain biomass was 15.5%.
Dry Stalk Biomass	kg/ha	[Stalk Biomass (g/plant) × Standing Plants]/Plot Size (hectare)	Average of five plants from the center of row cut at ground level at R6 growth stage, weighed, shredded, subsample weighed fresh and dry.
Harvest Index	/	Dry Grain Biomass (g/plant)/[Dry Stalk Biomass (g/plant) + Cob Biomass (g/plant) + Dry Grain Biomass (g/plant)]	/
Grain Density	/	/	Measured with a near-infrared (NIR) spectroscopy Perten DA7200 analyzer (Perten Instruments, Springfield, IL, USA) on kernels sampled five ears each plot.
Grain Nitrogen Content	kg/ha	[Grain Protein (%)/6.25] × Dry Grain Biomass (g/plant)]/Plot Size (hectare)	/
Grain Nitrogen Utilization Efficiency (Grain NutE)	/	Dry Grain Biomass (g/plant)/[Stalk N (%) × Stalk Biomass (g/plant) + [Grain Protein (%)/6.25] × Dry Grain Biomass (g/plant)]	Describe how the plant uses the nitrogen it acquires to produce grain. It is the ratio between dry grain biomass over the total Nitrogen content of the plant.
Plant Nitrogen Content	kg/ha	[Stalk N (%) × Stalk Biomass (g/plant) + [Grain Protein (%)/6.25] × Dry Grain Biomass (g/plant)]/Plot Size (hectare)	The amount of nitrogen of all standing plants normalized to their plot area. The total amount of nitrogen of each plant was the addition of the amount in stalk and in grain. The stalk nitrogen content was measured by a combustion analysis of dry stover. Grain protein percent was determined by a lab-based NIR spectrometer, which is converted to grain nitrogen content at the Jones factor of 6.25 in maize [52].

**Table 2:** A summary of UAV platforms with multiple aerial remote sensors and properties.

UAV Platform	Data Format	Sensor	Stabilizer	Recorded Information	Spectral Properties	GSD
DJI M600 Pro hexacopter (DJI Corporation, Shenzhen, China),	Hyperspectral Imagery	Headwall Nano-Hyperspec	DJI Ronin MX gimbal	270 VNIR spectral bands	400–1000 nm with FWHM of 6 nm	3 cm
	FLIR Thermal Imagery	FLIR Vue Pro R 640		/	/	/
	GPS/IMU	Applanix APX-15				
DJI M600 Pro hexacopter (DJI Corporation, Shenzhen, China),	LiDAR point cloud	Velodyne HDL-32	Hard mount	LiDAR point cloud and attributes	/	900 pts/m^2^
	RGB Imagery	Sony A7R II		Blue, Green, Red bands		2.4 cm
DJI M600 Pro hexacopter (DJI Corporation, Shenzhen, China),	ICI Thermal Imagery	ICI 8640 P-series	Gremsy T3 gimbal	1 thermal IR band	7–14 μm	8 cm
	RGB Imagery	Sony RX10				
	Multispectral Imagery	Micasense Altum	Hard mount	5 spectral bands: Blue, Green, Red, Red-edge, NIR		

**Table 3:** A summary of descriptive statistics of each maize phenotype collected at the end of growing season.

Phenotypes	Count	Mean	Std *	cv (%) **	Min	25%	50%	75%	Max
Dry Stalk Biomass (kg/ha)	369	6510.82	2153.74	33.1	1477	5033	6315	7756	22,035
Cob Biomass (kg/ha)	369	1470.71	498.90	33.9	415	1091	1432	1822	3853
Dry Grain Yield (kg/ha)	369	7176.92	3300.98	46	425	4282	7038	9848	17,450
Harvest Index	369	0.45	0.09	19.4	0.03	0.40	0.46	0.52	0.75
Grain NutE	369	55.92	11.10	19.9	5	50	57	63	77
Grain N (kg/ha)	369	91.70	49.48	53.9	9	44	90	136	218
Total Plant N (kg/ha)	369	135.88	70.18	51.7	26	68	141	198	314
Grain Density	369	1.27	0.038	3	1.02	1.25	1.27	1.3	1.35

* standard of deviation, ** coefficient of variation.

**Table 4:** Selected vegetation indices (VIs) across data modalities.

No.	Vegetation Index	Acronym	Equation	References
Hyperspectral-derived metrics				
1	Anthocyanin (Gitelson)	Ant_Gitelson_	Ant_Gitelson_ = (1/R_550_ − 1/R_700_ ) × R_780_	[70]
2	Chlorophyll Index	CI	CI = (R_750_ − R_ 705 _ )/(R_750_ + R_705_)	[71]
3	Optimized Soil-Adjusted Vegetation Index	OSAVI	OSAVI = (1 + 0.16) × (R_800_ –R _ 670 _ )/(R_800_ + R_670_ + 0.16)	[72]
4	Red Green Index	RGI	RGI = R_690_/R_550_	[73]
5	Structure Intensive Pigment Index	SIPI	SIPI = (R_800_ − R _ 450 _ )/(R_800_ + R_650_)	[74]
6	Transformed Chlorophyll Absorption in Reflectance Index	TCARI	TCARI = 3 × ((R_700_ − R _670_)− 0.2 × R_700_− R _550_)× (R_700_/R_670_))	[75]
7	Nitrogen Reflectance Index (NRI)	NRI	NRI = (R_570_ − R_670_)/(R_570_ + R_670_)	[76]
8	Modified Chlorophyll Absorption in Reflectance Index	mCARI	mCARI = 1.2 × (2.5 × (R_761_ − R _ 651 _ )–1.3 × (R_761_ − R _ 581 _ ))	[77]
9	Photochemical Reflectance Index	PRI	PRI = (R_531_ –R _ 570 _ )/(R_531_ + R_570_)	[78]
10	Ratio Analysis of reflectance Spectral Chlorophyll a	RARSa	RARSa = R_675_/R_700_	[79]
11	Ratio Analysis of reflectance Spectral Chlorophyll b	RARSb	RARSb = R_675_/(R_700_ × R_650_)	[79]
12	Ratio Analysis of reflectance Spectral	RARSc	RARSc = R_760_/R_500_	[79]
13	Pigment specific simple ratio	PSSR	PSSR = R_800_/R_680_	[80]
14	Plant Senescence Reflectance Index	PSRI	PSRI = (R_660_ − R_ 510_)/R_760_	[81]
15	Normalized chlorophyll pigment ratio index	NCPI	NCPI = (R_670_ − R_ 450_)/(R_670_ + R_450_)	[74]
16	Plant Pigment ratio	PPR	PPR = (R_550_ − R _ 450 _ )/(R_550_ + R_450_)	[82]
17	Normalized Difference Vegetation Index	NDVI	NDVI = (R_860_ − R _ 670 _ )/(R_860_ + R_670_)	[83]
18	Greenness Index	GI	GI = R_554_/R_677_	[73]
19	Green NDVI	GNDVI	GNDVI = (R_750_ − R _ 540 _ + R_570_)/(R_750_ + R_540_ − R _ 570 _ )	[84]
20	Simple Ratio	SR	SR = R_900_/R_680_	[85]
21	Red-edgeNDVI	RNDVI	RNDVI = (R_750_ − R_705_)/(R_750_ + R_705_)	[86]
22	Modified Triangular Vegetation Index	MTVI	MTVI = 1.2 × (1.2 × (R_800_ – R_550_) − 2.5 × (R_670_ − R _ 550 _ ))	[77]
23	Triangular Vegetation Index	TVI	TVI = 0.5 × (120 × (R_761_ − R _ 581 _ ) – 200(R_651_ − R _ 581 _ ))	[87]
24	Fluorescence Ratio Index 1	FRI_1_	FRI1 = R_690_/R_630_	[88]
25	Fluorescence Ratio Index 2	FRI_2_	FRI2 = R_750_/R_800_	[89]
26	Fluorescence Ratio Index 3	FRI_3_	FRI3 = R_690_/R_600_	[90]
27	Fluorescence Ratio Index 4	FRI_4_	FRI4 = R_740_/R_800_	[90]
28	Fluorescence Curvature Index	FCI	FCI = *R*^2^_683_/(R_675_ × R_691_)	[88]
29	Modified Red Edge Simple Ratio Index	mRESR	mRESR = (R_750_ − R _ 445 _ )/(R_705_ + R_445_)	[91]
30	Normalized Phaeophytinization Index	NPQI	NPQI = (R_415_ − R _ 435 _ )/(R_415_ + R_435_)	[92]
31	Red-Edge Vegetation Stress Index 1	RVS1	RVS1 =((R_651_ + R_750_)/2) − R_733_	[93]
32	Red-Edge Vegetation Stress Index 2	RVS2	RVS2 =((R_651_ + R_750_)/2) − R_751_	[93]
33	Water Index	WI	WI = R_900_/R_970_	[94]
34	Water Stress and Canopy Temperature	WSCT	WSCT = (R_970_ − R _ 850 _ )/(R_970_ + R_850_)	[95]
LiDAR-derived canopy height metrics				
1	Maximum of canopy height	Hmax		
2	Minimum of canopy height	Hmin		
3	Mean of canopy height	Hmean		
4	Mode of canopy height	Hmode		
5	Standard deviation of canopy height	Hsd		
6	Coefficient of variation of canopy height	Hcv		
7		Hmad	Hmad = 1.4826 × median (|height − Hmedian|)	
8		Haad	Haad = mean (|height − Hmean|)	
9–20	Percentile of canopy height	Hper	H10, H20, H30, H40, H50, H60, H70, H80, H90, H95, H98, H99	
21	The Interquartile Range (iqr) of canopy height	Hiqr	Hiqr = H75 − H25	
22	Skewness of canopy height	Hskn		
23	Kurtosis of canopy height	Hkurt		
24–28	Canopy return density of height	Hcrd	The proportion of points above the height quantiles (10th, 30th, 50th, 70^th^, and 90th) to the total number of points: Hd10, Hd30, Hd50, Hd70, Hd90	
29	Canopy relief ratio of height	Hcrr	(Hmean-Hmin)/(Hmax−Hmin)	
30		Hcg	The ratio of canopy returns of height and ground returns of height	
LiDAR-derived canopy intensity metrics				
1	Maximum of canopy intensity	Imax		
2	Minimum of canopy intensity	Imin		
3	Mean of canopy intensity	Imean		
4	Mode of canopy intensity	Imode		
5	Standard deviation of canopy intensity	Isd		
6	Coefficient of variation of canopy intensity	Icv		
7		Imad	Imad = 1.4826 × median (|intensity − Imedian|)	
8		Iaad	Iaad = mean (|intensity−Imean|)	
9–20	Percentile of canopy intensity	Iper	I10, I20, I30, I40, I50, I60, I70, I80, I90, I95, I98, I99	
21	The Interquartile Range (iqr) of canopy intensity	Iiqr	Iiqr = I75−I25	
22	Skewness of canopy intensity	Iskn		
23	Kurtosis of canopy intensity	Ikurt		
24–28	Canopy return density of intensity	Icrd	The proportion of points above the intensity quantiles (10th, 30th, 50th, 70th, and 90th) to the total number of points: Id10, Id30, Id50, Id70, Id90	
29	Canopy relief ratio of intensity	Icrr	(Imean–Imin)/(Imax−Imin)	
30		Icg	The ratio of canopy returns of intensity and ground returns of intensity	
Thermal-derived metric				
1	Normalized relative canopy temperature index	Tir	Tir = (Ti–Tmin)/(Ti–Tmax)	[96]

**Figure A1:**
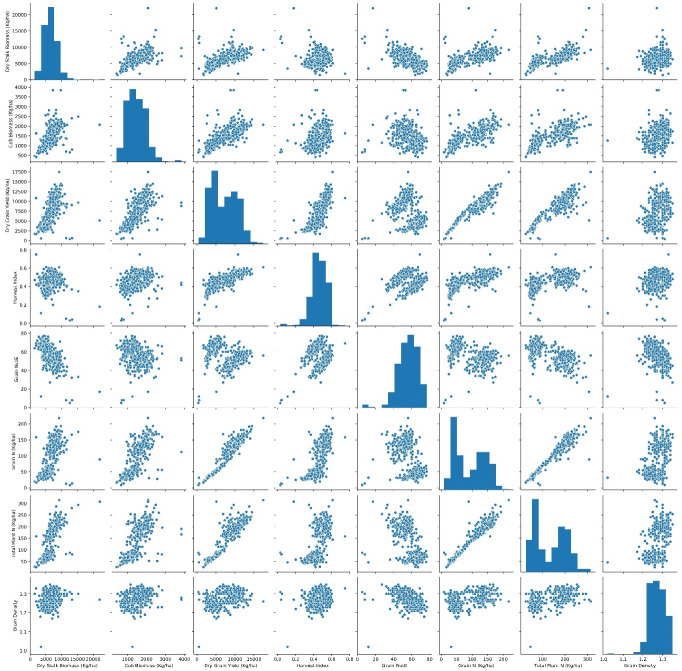
Histograms of Maize Phenotypes (In Diagonal) and Scatter Plots between Pairs of the Phenotypes.

## Data Availability

Not applicable.

## Appendix B

**Table A1:** Results of maize phenotypic prediction performed by different data sources and regressors.

Datasets	Metrics	Stalk Biomass (kg/ha)								Cob Biomass (kg/ha)								Dry Grain Yield (kg/ha)							
		Hand-Crafted Features-Based				Imagery-Based				Hand-Crafted Features-Based				Imagery-Based				Hand-Crafted Features-Based				Imagery-Based			
		SVR		RFR		Mono-Task		Multi-Task		SVR		RFR		Mono-Task		Multi-Task		SVR		RFR		Mono-Task		Multi-Task	
		Train	Test	Train	Test	Train	Test	Train	Test	Train	Test	Train	Test	Train	Test	Train	Test	Train	Test	Train	Test	Train	Test	Train	Test
Thermal	*R* ^2^	0.06	0	0.1	0.02	0.15	0.12	0.15	0.11	0.05	0	0.08	0	0.22	0.22	0.2	0.2	0.07	0.02	0.13	0.05	0.28	0.26	0.28	0.26
	*MAE*	1522	1518	1519	1486	1419	1403	1422	1404	377	401	377	393	336	335	339	337	2672	2784	2603	2743	2272	2351	2273	2326
	*RMSE*	2120	1969	2088	1943	2015	1844	2016	1855	485	507	474	498	438	443	444	448	3170	3252	3081	3211	2786	2833	2795	2830
LiDAR Intensity	*R* ^2^	0.2	0.09	0.16	0.04	0.15	0.16	0.14	0.13	0.3	0.09	0.16	0.03	0.19	0.22	0.18	0.21	0.37	0.04	0.17	0	0.14	0.17	0.13	0.15
	*MAE*	1394	1440	1463	1502	1454	1355	1473	1360	316	363	362	383	352	341	356	346	2108	2615	2560	2807	2484	2462	2510	2493
	*RMSE*	1961	1867	2003	1926	2019	1805	2034	1824	416	474	454	492	446	443	449	446	2603	3209	2993	3311	3047	3008	3065	3023
LiDAR Height	*R* ^2^	0.55	0.48	0.58	0.45	0.37	0.38	0.31	0.31	0.37	0.27	0.45	0.33	0.28	0.3	0.29	0.30	0.47	0.33	0.41	0.37	0.25	0.25	0.26	0.26
	*MAE*	996	1040	1052	1108	1254	1191	1326	1266	276	307	274	300	325	313	323	313	1698	1886	1925	1956	2269	2249	2254	2250
	*RMSE*	1472	1414	1419	1464	1744	1550	1823	1632	393	423	369	410	422	419	419	418	2390	2668	2526	2611	2857	2851	2843	2840
Hyper spectral	*R* ^2^	0.54	0.5	0.48	0.36	0.43	0.36	0.4	0.32	0.57	0.5	0.56	0.46	0.45	0.37	0.45	0.39	0.81	0.77	**0.81**	**0.78**	0.76	0.72	0.76	0.73
	*MAE*	953	1000	1076	1146	1131	1167	1145	1171	223	244	238	262	270	291	268	284	1061	1206	**1082**	**1175**	1225	1323	1218	1276
	*RMSE*	1488	1392	1582	1576	1651	1568	1703	1613	324	353	329	368	369	398	368	390	1430	1578	**1438**	**1554**	1602	1722	1616	1688
Hyper + LiDAR Height	*R* ^2^	**0.6**	**0.53**	0.64	0.47	0.5	0.46	0.4	0.37	0.54	0.43	**0.58**	**0.47**	0.47	0.4	0.44	0.41	0.84	0.73	0.81	0.78	0.76	0.73	0.73	0.72
	*MAE*	**911**	**1008**	989	1068	1069	1096	1164	1156	221	265	**233**	**259**	264	281	271	279	914	1297	1083	1176	1223	1287	1274	1312
	*RMSE*	**1375**	**1343**	1321	1428	1545	1443	1694	1549	335	376	**322**	**364**	363	387	372	384	1301	1682	1437	1555	1609	1691	1708	1718
Hyper + LiDAR Height + LiDAR Intensity	*R* ^2^	0.57	0.49	0.64	0.47	0.47	0.47	0.42	0.39	0.48	0.41	0.57	0.47	0.44	0.41	0.45	0.41	0.86	0.72	0.81	0.77	0.73	0.72	0.74	0.72
	*MAE*	946	1028	990	1069	1116	1088	1147	1135	248	271	236	261	272	281	267	279	833	1380	1082	1179	1282	1323	1268	1315
	*RMSE*	1437	1393	1321	1430	1594	1430	1674	1530	357	383	326	365	372	386	368	384	1197	1748	1436	1558	1705	1730	1692	1723
Hyper + LiDAR Height + LiDAR Intensity+ Thermal	*R* ^2^	0.57	0.49	0.64	0.47	0.47	0.46	0.45	0.43	0.48	0.41	0.57	0.46	0.44	0.40	0.46	0.43	0.85	0.72	0.81	0.77	0.74	0.72	0.73	0.71
	*MAE*	939	1038	989	1070	1113	1083	1107	1108	248	271	237	263	269	284	262	274	923	1361	1082	1179	1277	1338	1267	1321
	*RMSE*	1432	1402	1321	1430	1599	1448	1623	1478	357	383	326	367	370	388	365	380	1228	1734	1436	1558	1696	1752	1701	1747

**Table A2:** Results of maize phenotypic prediction performed by different data sources and regressors (cont.).

Datasets	Metrics	Harvest Index								Grain Nitrogen Utilization Efficiency (Grain NutE)								Grain Nitrogen Content (kg/ha)							
		Hand-Crafted Features-Based				Imagery-Based				Hand-Crafted Features-Based				Imagery-Based				Hand-Crafted Features-Based				Imagery-Based			
		SVR		RFR		Mono-Task		Multi-Task		SVR		RFR		Mono-Task		Multi-Task		SVR		RFR		Mono-Task		Multi-Task	
		Train	Test	Train	Test	Train	Test	Train	Test	Train	Test	Train	Test	Train	Test	Train	Test	Train	Test	Train	Test	Train	Test	Train	Test
Thermal	*R* ^2^	0.02	0	0.07	0	0.14	0.13	0.13	0.13	0	0	0.04	0	0.12	0.1	0.11	0.09	0.03	0	0.12	0.03	0.33	0.3	0.33	0.31
	*MAE*	0.07	0.07	0.07	0.07	0.06	0.06	0.06	0.06	8.3	7.97	8.42	8.17	7.78	7.41	7.78	7.43	41.08	43.28	40.6	43.28	33.41	34.7	33.22	34.66
	*RMSE*	0.09	0.09	0.09	0.09	0.08	0.08	0.08	0.08	11.14	10.89	10.94	10.76	10.49	10.15	10.53	10.24	48.44	50.67	46.29	48.97	40.25	41.34	40.33	41.30
LiDAR Intensity	*R* ^2^	0.12	0	0.1	0	0.04	0.04	0.02	0.02	0.14	0.08	0.17	0.02	0.11	0.11	0.09	0.09	0.38	0.05	0.2	0.02	0.19	0.2	0.17	0.18
	*MAE*	0.06	0.07	0.07	0.07	0.07	0.06	0.07	0.07	7.76	7.58	7.81	7.91	7.89	7.64	8.08	7.66	31.29	39.66	39.14	43.55	37.98	38.0	38.94	39.1
	*RMSE*	0.08	0.09	0.08	0.09	0.09	0.09	0.09	0.09	10.36	10.27	10.15	10.60	10.52	10.14	10.69	10.22	38.58	48.06	44.09	49.09	44.39	44.26	44.99	45.04
LiDAR Height	*R* ^2^	0.23	0.02	0.28	0.12	0.05	0.04	0.04	0.03	0.33	0.2	0.35	0.26	0.18	0.17	0.17	0.19	0.54	0.36	0.47	0.42	0.3	0.3	0.3	0.3
	*MAE*	0.05	0.06	0.06	0.06	0.07	0.06	0.07	0.06	6.48	6.96	6.81	6.96	7.52	7.37	7.63	7.41	23.24	27.93	27.35	28.63	33.98	34.33	34.26	34.53
	*RMSE*	0.08	0.09	0.08	0.08	0.09	0.09	0.09	0.09	9.15	9.59	9.03	9.24	10.12	9.74	10.16	9.65	33.27	39.52	35.74	37.60	41.1	41.55	41.31	41.57
Hyper spectral	*R* ^2^	0.53	0.49	0.53	0.42	0.45	0.41	0.45	0.41	0.39	0.29	0.44	0.3	0.36	0.29	0.33	0.27	0.87	0.82	**0.88**	**0.85**	0.85	0.81	0.84	0.81
	*MAE*	0.04	0.04	0.04	0.04	0.04	0.04	0.04	0.04	5.57	5.85	5.86	6.01	6.13	6.05	6.23	6.15	12.79	15.87	**12.67**	**13.97**	14.66	16.13	14.69	16.33
	*RMSE*	0.06	0.06	0.06	0.07	0.07	0.07	0.07	0.07	8.75	9.05	8.35	8.97	8.92	9.04	9.13	9.18	17.66	20.95	**17.22**	**19.24**	19.26	21.37	19.63	21.56
Hyper + LiDAR Height	*R* ^2^	0.55	0.48	**0.6**	**0.56**	0.47	0.41	0.42	0.43	0.42	0.32	**0.55**	**0.49**	0.38	0.3	0.27	0.25	0.87	0.81	0.88	0.85	0.84	0.81	0.81	0.79
	*MAE*	0.04	0.04	**0.04**	**0.04**	0.04	0.04	0.05	0.04	5.36	5.90	**5.51**	**5.55**	6.01	6.12	6.74	6.51	12.69	16.62	12.67	13.99	14.63	16.21	16.03	16.77
	*RMSE*	0.06	0.06	**0.06**	**0.06**	0.06	0.07	0.07	0.07	8.48	8.82	**7.48**	**7.62**	8.79	8.97	9.54	9.27	17.77	21.76	17.22	19.27	19.58	21.73	21.42	22.43
Hyper + LiDAR Height + LiDAR Intensity	*R* ^2^	0.56	0.47	0.6	0.55	0.46	0.42	0.42	0.43	0.44	0.33	0.56	0.48	0.26	0.24	0.27	0.27	0.85	0.79	0.88	0.85	0.81	0.79	0.81	0.79
	*MAE*	0.04	0.04	0.04	0.04	0.04	0.04	0.04	0.04	5.37	6.02	5.55	5.57	6.86	6.67	6.71	6.44	14.4	16.71	12.66	14.00	15.99	16.96	15.86	16.89
	*RMSE*	0.06	0.06	0.06	0.06	0.07	0.07	0.07	0.07	8.34	8.77	7.43	7.70	9.62	9.37	9.53	9.17	19.33	22.36	17.21	19.27	21.25	22.62	21.22	22.65
Hyper + LiDAR Height + LiDAR Intensity + Thermal	*R* ^2^	0.55	0.46	0.6	0.55	0.45	0.42	0.44	0.45	0.45	0.31	0.56	0.48	0.29	0.23	0.29	0.29	0.86	0.8	0.88	0.85	0.81	0.78	0.82	0.79
	*MAE*	0.04	0.04	0.04	0.04	0.04	0.04	0.04	0.04	5.21	6.08	5.55	5.57	6.71	6.65	6.61	6.36	13.47	16.65	12.66	14.00	16.24	17.01	15.51	16.7
	*RMSE*	0.06	0.06	0.06	0.06	0.07	0.07	0.07	0.07	8.24	8.90	7.43	7.69	9.44	9.39	9.39	9.02	18.57	22.20	17.21	19.28	21.66	23.10	21.15	22.50

**Table A3:** Results of maize phenotypic prediction performed by different data sources and regressors (cont.).

Datasets	Metrics	Total Plant N (kg/ha)								Grain Density							
		Hand-Crafted Features-Based				Imagery-Based				Hand-Crafted Features-Based				Imagery-Based			
		SVR		RFR		Mono-Task		Multi-Task		SVR		RFR		Mono-Task		Multi-Task	
		Train	Test	Train	Test	Train	Test	Train	Test	Train	Test	Train	Test	Train	Test	Train	Test
Thermal	*R* ^2^	0.03	0	0.13	0.05	0.34	0.32	0.32	0.29	0.01	0	0.03	0	0.14	0.09	0.12	0.11
	*MAE*	58.2	60.79	57.97	60.30	46.8	47.91	48.32	49.19	0.03	0.03	0.03	0.03	0.03	0.02	0.03	0.03
	*RMSE*	68.96	71.64	65.48	68.07	56.93	57.49	57.74	58.52	0.04	0.04	0.04	0.04	0.04	0.04	0.04	0.03
LiDAR Intensity	*R* ^2^	0.42	0.04	0.21	0.04	0.21	0.23	0.18	0.19	0.11	0.05	0.13	0.03	0.1	0.12	0.1	0.13
	*MAE*	43.12	54.85	55.63	61.01	52.5	51.9	55.19	54.67	0.03	0.03	0.03	0.03	0.03	0.02	0.03	0.03
	*RMSE*	53.19	67.95	62.47	68.23	62.17	60.97	63.65	62.65	0.04	0.04	0.04	0.04	0.04	0.03	0.04	0.03
LiDAR Height	*R* ^2^	0.58	0.44	0.52	0.47	0.36	0.36	0.34	0.34	0.29	0.15	0.28	0.19	0.14	0.13	0.15	0.14
	*MAE*	32.49	37.95	37.19	39.17	46.26	46.61	46.77	47.39	0.02	0.03	0.02	0.02	0.03	0.03	0.03	0.03
	*RMSE*	45.58	51.94	48.53	50.80	56.26	55.94	56.86	56.57	0.03	0.03	0.03	0.03	0.04	0.03	0.04	0.03
Hyperspectral	*R* ^2^	0.88	0.85	0.88	0.86	0.87	0.85	0.85	0.83	0.4	0.29	0.38	0.28	0.34	0.32	0.33	0.32
	*MAE*	17.37	20.39	17.31	18.98	18.97	20.25	20.01	21.56	0.02	0.02	0.02	0.02	0.02	0.02	0.02	0.02
	*RMSE*	24.51	26.91	24.24	26.16	25.45	27.06	27.18	28.74	0.03	0.03	0.03	0.03	0.03	0.03	0.03	0.03
Hyper + LiDAR Height	*R* ^2^	0.88	0.85	**0.88**	**0.85**	0.86	0.84	0.82	0.82	0.47	0.34	**0.44**	**0.34**	0.34	0.32	0.31	0.31
	*MAE*	17.27	20.38	**17.41**	**19.16**	19.68	20.83	22.25	22.62	0.02	0.02	**0.02**	**0.02**	0.02	0.02	0.02	0.02
	*RMSE*	24.3	27.22	**24.15**	**26.64**	26.25	27.43	29.53	29.40	0.03	0.03	**0.03**	**0.03**	0.03	0.03	0.03	0.03
Hyper + LiDAR Height + LiDAR Intensity	*R* ^2^	0.87	0.83	0.88	0.85	0.84	0.83	0.83	0.82	0.38	0.31	0.46	0.35	0.3	0.28	0.3	0.3
	*MAE*	17.9	21.82	17.39	19.22	21.37	22.24	21.98	22.5	0.02	0.02	0.02	0.02	0.03	0.03	0.03	0.02
	*RMSE*	24.86	28.31	24.11	26.74	28.4	28.68	29.17	29.37	0.03	0.03	0.03	0.03	0.03	0.03	0.03	0.03
Hyper + LiDAR Height + LiDAR Intensity + Thermal	*R* ^2^	0.88	0.83	0.88	0.85	0.83	0.83	0.83	0.82	0.38	0.32	0.46	0.35	0.29	0.25	0.31	0.29
	*MAE*	17.12	21.93	17.39	19.23	21.63	21.85	21.65	22.65	0.02	0.02	0.02	0.02	0.03	0.03	0.02	0.02
	*RMSE*	23.95	28.54	24.11	26.75	28.76	28.39	28.94	29.40	0.03	0.03	0.03	0.03	0.03	0.03	0.03	0.03
